# Inhibition of CSPG receptor PTPσ promotes migration of newly born neuroblasts, axonal sprouting, and recovery from stroke

**DOI:** 10.1016/j.celrep.2022.111137

**Published:** 2022-07-26

**Authors:** Fucheng Luo, Jiapeng Wang, Zhen Zhang, Zhen You, Alicia Bedolla, FearGod Okwubido-Williams, L. Frank Huang, Jerry Silver, Yu Luo

**Affiliations:** 1 Department of Molecular Genetics, Biochemistry, and Microbiology, College of Medicine, University of Cincinnati, Cincinnati, OH 45229, USA; 2 Department of Pharmaceutical Sciences, College of Pharmacy, University of Cincinnati, Cincinnati, OH 45229, USA; 3 Division of Experimental Hematology and Cancer Biology, Brain Tumor Center, Cincinnati Children’s Hospital Medical Center, Cincinnati, OH 45229, USA; 4 Department of Pediatrics, College of Medicine, University of Cincinnati, Cincinnati, OH 45221, USA; 5 Department of Neurosciences, Case Western Reserve University, Cleveland, OH 44106, USA; 6 Lead contact

## Abstract

In addition to neuroprotective strategies, neuroregenerative processes could provide targets for stroke recovery. However, the upregulation of inhibitory chondroitin sulfate proteoglycans (CSPGs) impedes innate regenerative efforts. Here, we examine the regulatory role of PTPσ (a major proteoglycan receptor) in dampening post-stroke recovery. Use of a receptor modulatory peptide (ISP) or *Ptprs* gene deletion leads to increased neurite outgrowth and enhanced NSCs migration upon inhibitory CSPG substrates. Post-stroke ISP treatment results in increased axonal sprouting as well as neuroblast migration deeply into the lesion scar with a transcriptional signature reflective of repair. Lastly, peptide treatment post-stroke (initiated acutely or more chronically at 7 days) results in improved behavioral recovery in both motor and cognitive functions. Therefore, we propose that CSPGs induced by stroke play a predominant role in the regulation of neural repair and that blocking CSPG signaling pathways will lead to enhanced neurorepair and functional recovery in stroke.

## In brief

ECM molecules called chondroitin sulfate proteoglycans are barriers to axonal plasticity and precursor cell migration after stroke. Luo et al. use a peptide to block PTPσ, a receptor that mediates the inhibition. Peptide treatment results in significant behavioral recovery accompanied by neuroprotection, axonal sprouting, and neuroblast migration into the lesion.

## Graphical Abstract

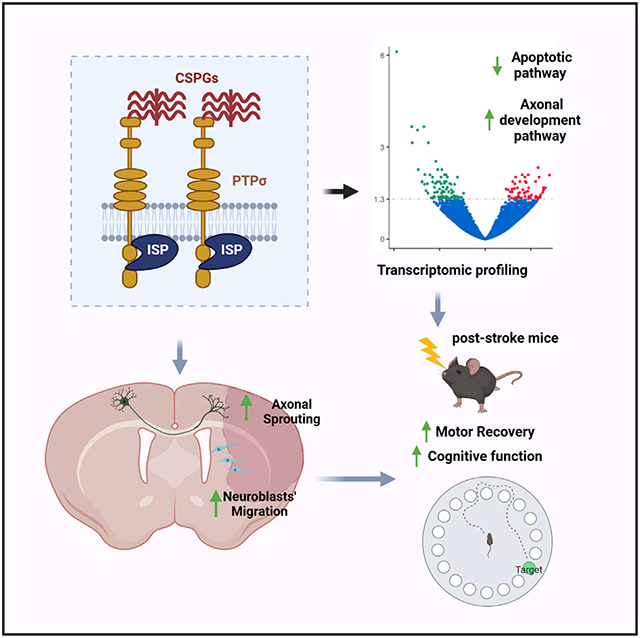

## INTRODUCTION

Stroke profoundly alters the lives of affected individuals and is one of the leading causes of death and disability worldwide (Benjamin et al., 2017). Current treatment strategies are largely neuroprotective and all are limited by narrow time windows (Gilman, 2006; Goldstein, 2007). However, the potential for regeneration/plasticity in the post-stroke CNS is still possible for weeks or even longer, which may provide an extended opportunity for treatment (Zhang et al., 2016). Two potential processes for repair are axonal sprouting and neurogenesis (Carmichael, 2008; Wiersma et al., 2017). Understanding how these endogenous mechanisms may be further stimulated to contribute to recovery will help in the development of novel therapeutic interventions.

Ablation studies have suggested that newly born neuroblasts may contribute to functional recovery after stroke, despite the low numbers that can survive as maturing neurons (Jin et al., 2010; Sun et al., 2012). Although stroke stimulates this process, the endogenous response is inadequate (Chopp and Li, 2008). Due to the hostile environment in the damaged brain, many of the newly born neurons approach but cannot invade the stroke peri-infarct region to intermingle with surviving neuropil and they mostly die within 1 week after their birth (Dempsey et al., 2003). This indicates the need for strategies that can enhance both the survival and migration of newly born neuroblasts. Stroke also induces axonal sprouting in the brain in the form of new ipsilateral local circuits as well as expanded intercortical connections and descending projection reorganizations (Wiersma et al., 2017). However, sprouting is also insufficient in the lesioned mammalian CNS. One critical repair-limiting factor for both neurons and neural stem cells (NSCs) is the family of potently inhibitory ECM molecules known as chondroitin sulfate proteoglycans (CSPGs) (Tran et al., 2018b). Certain CSPGs are upregulated in abundance in glial scars after brain or spinal cord injury (SCI) (McKeon et al., 1991; Silver and Miller, 2004; Tran et al., 2018b). CSPGs in the scar limit regeneration through the lesion but they also severely restrain potential neuroplasticity around and beyond the lesion perimeter (Tran et al., 2022). CSPGs have also been suggested to curtail the access of progenitor cells to remyelinate cord and multiple sclerosis (MS) lesions (Dyck et al., 2015; Dyck and Karimi-Abdolrezaee, 2015; Kazanis and ffrench-Constant, 2011; Lau et al., 2012; Siebert and Osterhout, 2021).

In models of stroke (Carmichael, 2010; Chen et al., 2014; Soleman et al., 2012; Wiersma et al., 2017), chondroitinase ABC (ChABC) has been used therapeutically by targeted injection into the spinal cord. While results were encouraging, the effects were limited likely due to minimal spread of the enzyme. To overcome the limitations of native ChABC, several labs have shown successful long-term and/or widespread delivery and efficacy in stroke and SCI models using thermostabilized (Hettiaratchi et al., 2019, 2020; Lee et al., 2010) and viral-mediated formulations of chondroitinase (Bartus et al., 2014; Burnside et al., 2018), although the potential complications of direct *in vivo* administration remained.

The transmembrane receptor protein tyrosine phosphatase-sigma (PTPσ) has been identified as a major receptor for the inhibitory actions of CSPGs (Shen et al., 2009). To modulate proteoglycan-mediated inhibition over large regions, we have used systemic agents that could block chondroitin sulfate-glycosaminoglycan (CS-GAG) interactions with this receptor in the presence of any evolving lesion without the need to directly impale the CNS parenchyma. Intracellular sigma peptide (ISP), a peptide mimetic of the PTPσ regulatory wedge region with a TAT domain to facilitate membrane and CNS penetration (Lang et al., 2015), was designed for this purpose. ISP has very high specificity for PTPσ (Sakamoto et al., 2019). Importantly, after systemic delivery, ISP rapidly enters the CNS and leads to significant axonal sprouting with restored sensory motor and bladder function after acute contusive cord injury in adult rats (Lang et al., 2015; Rink et al., 2018) and has also led to the enhanced migration, differentiation, and remyelination by oligodendrocyte precursor cells (OPCs) with functional recovery in mouse models of MS (Luo et al., 2018) and SCI (Dyck et al., 2018, 2019).

Whether CSPG-PTPσ signaling may play a role in a large injury such as stroke has not been investigated. By genetically and pharmacologically restricting the inhibitory properties of sulfated proteoglycans, we investigated the untoward effects of CSPGs on two major neurorepair mechanisms—axonal sprouting and the generation of new neuroblasts as well as their migration after stroke. We also explored the potential molecular pathways through which CSPGs modulate adult NSC biology.

## RESULTS

### CSPGs are upregulated in the glial scar after stroke

**Figure 1.**
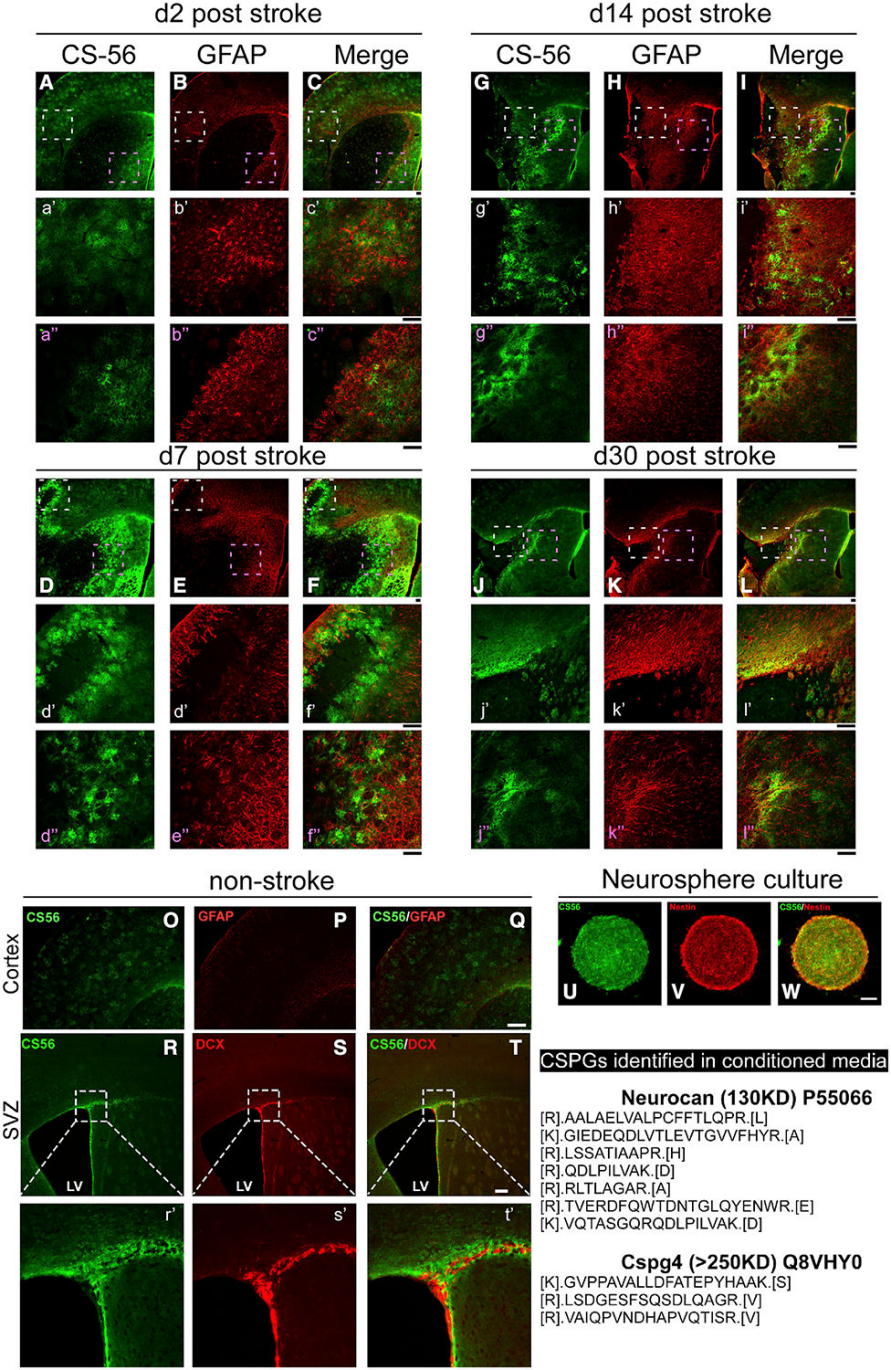
Accumulation of CSPGs near the infarct border at 2–30 days after stroke and NSCs (A–L) CSPG staining in stroke brains. Higher magnification (a’–l”). Representative images are shown with >3 mice with similar results for each time point. (M–R) CSPGs staining in non-stroke brain within the cortex or SVZ niche (higher magnification inp’-r’). (S–U) CSPGs are present in *ex vivo* adult SVZ neurospheres. Lower right panel shows CSPGs detected by mass spectrometry in conditioned media from neurosphere cultures (see Table S1 for full list). Scale bar: 50 μm.

To examine whether CSPGs are enriched in the glial scar after ischemic stroke, we stained sections containing the infarct with the CS56 antibody during the acute (2 days), subacute (7 days), and more chronic stages (14 and 30 days) post-stroke. Indeed, Figures 1A-1L show that CSPGs are enriched near the border of the lesion both in the cortex (white dashed box with higher magnification images in a’–l’) and striatum (pink dashed box with higher magnification images in a”–il’) compared to levels in the non-stroke brain (Figures 1M-1O), with peak upregulation at day 7 but sustained until day 30, especially near the glial scar. The pattern of CS56 staining in the lesion penumbra is consistent with the CSPG accumulation that occurs in humans (Huang et al., 2014) and in scar astrocytes described previously (Okuda, 2018) and with the observations of others who have described CSPG upregulation in the stroke penumbra of rodents (Carmichael, 2010; Gherardini et al., 2015). This indicates that CSPGs may play a role in stroke recovery.

### CSPGs are expressed in neurosphere cultures and enriched in the subventricular zone (SVZ) *in vivo*

The regulatory role of CSPGs in neuronal migration and axonal growth has been well established (Tran et al., 2018b); however, whether this family of extracellular matrix (ECM) molecules also plays a role in constraining the migration of adult neural progenitor cells after stroke is not known. We examined the expression patterns of CSPGs in cultured neurospheres derived from the adult SVZ (Figures 1S-1U), as well as *in vivo,* within neurogenic regions in 3-month-old mice (Figures 1P-1R). CSPGs are abundant in neurosphere cultures and in the SVZ where the adult neural stem cells reside (Gates et al., 1995). We detected CSPGs in the conditioned media derived from cultured neurospheres with mass spectrometry, suggesting that they produce and secrete CSPGs *in vitro* (full list of top proteins included in Table S1). Within the SVZ, the CS56 staining is evident at the laterodorsal corner of the lateral ventricle and extending along the length of the ventricular wall adjacent to the striatum. Co-immunostaining for doublecortin (DCX), a neuroblast and immature neuron marker, shows that the newly born neuroblasts in the SVZ are embedded within a CSPG-containing matrix. These results suggest that CSPGs could play a role in the regulation of adult neural stem cells in the SVZ and, importantly, that the NSCs themselves can produce a CSPG-laden matrix (Gates et al., 1995; Ida et al., 2006; Sirko et al., 2010).

### Inhibition of CSPG-PTPσ signaling leads to increased neurite outgrowth in SVZ NSC-differentiated neurons

**Figure 2.**
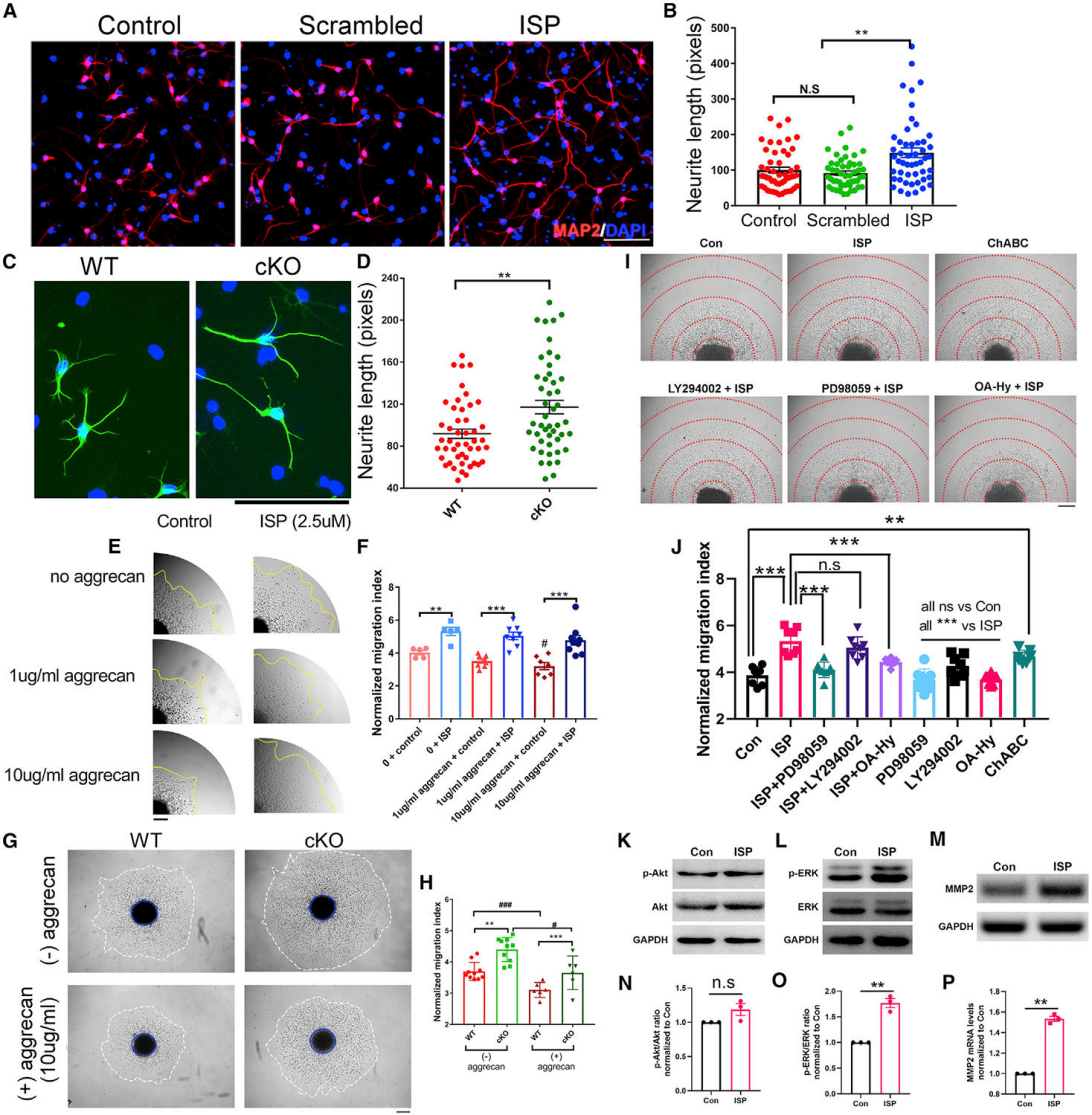
Inhibition of CSPG-PTPσ signaling leads to increased neurite outgrowth and migration of SVZ NSCs (A and B) Inhibition of PTPσ by ISP shows increased neurite outgrowth compared to controls. (C and D) Primary *Ptprs* cKO adult NSCs (AAV-Cre infected *Ptprs* floxed NSCs) also show increased neurite outgrowth compared to WT. Total of more than 50 cells were quantified from 3 tissue culture wells. Representative data shown from at least 3 independent experiments. (E and F) Increased CSPG concentrations lead to decreased migration of adult NSCs grown as neurospheres, and ISP leads to increased migration from SVZ neurospheres. (G and H) PTPσ deletion in adult NSCs also results in enhanced migration under both basal conditions and with additional CSPG coating (aggrecan 1 or 10 μg/mL). #, p < 0.05 or ###, p < 0.001 compared to no aggrecan; **p < 0.01 and ***p < 0.001 compared to control peptide or WT. (I and J) ISP enhances NSCs migration via disinhibition of the ERK pathway and upregulation of MMP2 activity. **p < 0.01 and ***p < 0.001. (K–P) ISP treatment of NSCs led to increased p-ERK levels, while it had no effect on pAkt levels. ISP increases *Mmp2* mRNA levels. **p < 0.01. For neurosphere migration assays, each data point represents 1 neurosphere, and data were pooled from 2–3 independent experiments. ANOVA for multiple group analysis and Student’s t test for 2 group analyses.

To test the regulatory role of the CSPG receptor, adult neural stem cells were dissociated and differentiated for 5 days in the presence of ISP. Inhibition of PTPσ by ISP led to increased neurite outgrowth in differentiated NSCs *in vitro,* while the scrambled peptide had no effect (Figures 2A and 2B). Interestingly, genetic knockout (KO) of RPTPσ in adult NSCs showed similar growth-promoting effects (Figures 2C and 2D), confirming and extending the previous work (Kirkham et al., 2006) that inhibition of PTPσ signaling enhances neurite outgrowth in adult differentiated NSC-derived neurons *in vitro.* Also, NSCs produce CSPGs themselves (Figure 1), which explains why the inhibition of PTPσ enhances NSC neurite elongation without an aggrecan substrate. Therefore, both pharmacological and genetic inhibition of the PTPσ receptor leads to increased neurite growth of adult NSCs *in vitro.*

### CSPG-PTPσ inhibition leads to enhanced migration of NSCs in the presence of exogenous CSPGs

Since neuroblast migration is critical during adult neurogenesis after CNS injury, we examined the movements of SVZ NSC-derived neuroblasts in the absence or presence of additional CSPGs. Individual neurospheres that were similar in size were picked from neurosphere cultures (days *in vitro* [DIV] 5–6) and plated upon different concentrations of aggrecan and the translocation of cells from the individual neurospheres was quantified by the migration index. CSPG-containing neurospheres treated with ISP showed increased migration on poly-L-lysine-coated culture surfaces (Figures 2E and 2F and Video S1 for time lapse of the migration). In the injured brain, because reactive astrocytes produce additional CSPGs within the substrate around the lesion (Filous and Silver, 2016), we also tested the migration of NSCs in the presence of an extra aggrecan substrate coating (Figures 2E and 2F). We observed further decreases in the migration of NSCs with increased aggrecan concentration, suggesting that CSPGs potently inhibit the migration of NSCs but, more interestingly, we observed the increased migration of NSCs with ISP treatment even with increasing aggrecan concentrations, demonstrating that ISP can reverse the inhibitory effects of CSPGs on adult NSC migration. To further validate our results, we also measured the migration of wild-type (WT) or *Ptprs* conditional KO (cKO) NSCs in the absence or presence of aggrecan (Figures 2G and 2H). To avoid potential compensatory effects during the development of the *Ptprs* KO, we cultured adult NSCs from floxed *Ptprs* animals and infected the WT cells and the floxed cells with AAV-CMV-Cre one passage before they were harvested for the neurosphere migration assay (deletion of the floxed *Ptprs* is validated in Figure S1). *Ptprs* KO NSCs demonstrated enhanced migration compared to WT NSCs both in the absence and presence of aggrecan (Figures 2G and 2H).

### ISP mediates enhanced migration of adult NSCs by modulating the ERK pathway and production of matrix metalloproteinase 2 (MMP2)

CSPG-PTPσ binding is known to modulate multiple signaling events in cells including pathways such as AKT and ERK as well as the recently identified ISP-induced production of the CSPG degrading enzyme, matrix metalloproteinase 2 (MMP2) (Luo et al., 2018; Ohtake et al., 2016). To examine the molecular pathways that play a role in mediating ISP-induced migration enhancement, we incubated neurospheres with control media, ChABC (5 mU/mL), ISP containing media (2.5 μM) alone or with inhibitors for the AKT pathway (LY294002, 10 μM), ERK pathway (PD 98059, 10 μM), and the MMP2 inhibitor (OA-Hy, 100 nM). We observed a similar effect of enhanced migration of neuroblasts out of neurospheres with ChABC treatment (Figures 2I and 2J), which further supports the role of CSPGs in the inhibition of adult NSC migration. Interestingly, the inhibition of ERK signaling reversed the effect of ISP on NSC migration, while the AKT inhibitor had no effect (Figures 2I and 2J). In addition, our results show that MMP2 inhibition is able to reverse the effect of ISP on NSC migration, consistent with our previous findings (Luo et al., 2018) that ISP increases MMP2 production in OPCs while expanding this mechanism to a new type of cell, adult NSC-derived neuroblasts (Figures 2I and 2J). Importantly, treatment with the specific inhibitors by themselves when ISP peptide was absent did not affect cell exodus from neurospheres (Figure 2J), suggesting that ERK inhibitors and MMP2 inhibitors specifically reverse the activation of ERK signaling and MMP2 upregulation caused by ISP treatment. Western blot and qRT-PCR analyses confirmed that ISP treatment in NSCs activates the phosphorylation of ERK (Figures 2L and 2O) without affecting p-Akt levels (Figures 2K and 2N). ISP treatment also increased the mRNA level of *Mmp2* (Figures 2M and 2P).

### Post-stroke ISP treatment enhances the number and migration of DCX^+^ neuroblasts into the stroke lesion epicenter

**Figure 3.**
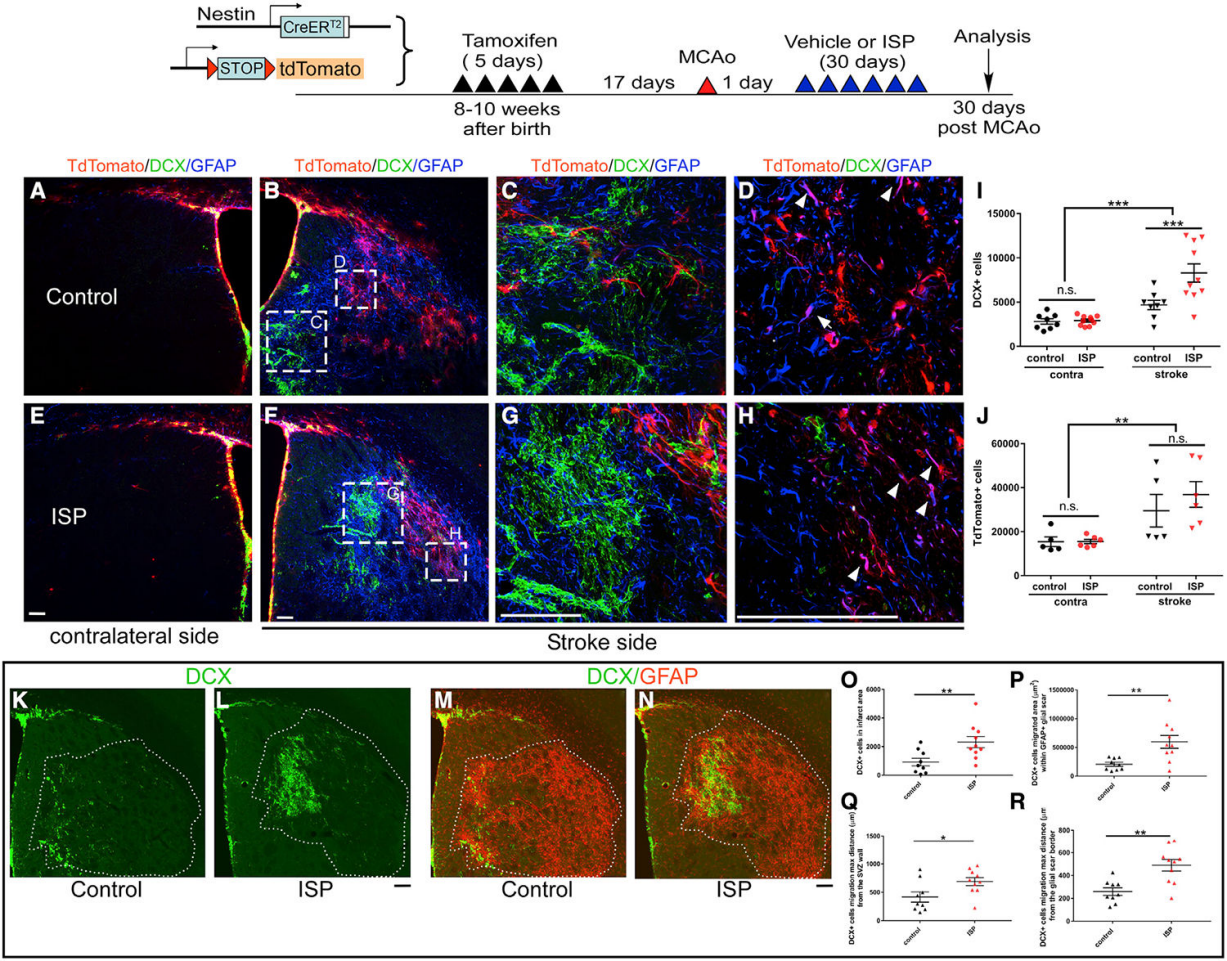
ISP treatment increased newly born neuroblast migration deep into the striatum 1 month after stroke For lineage tracing, Nestin creER-tdTomato mice were used. Stroke mice receiving vehicle (A–D, K, and M) or ISP treatment (E–H, L, and N) starting from day 1 post-stroke for 30 days. Contralateral side showing minimal migration of DCX^+^ neuroblasts (arrows) or tdTomato^+^ cells into the striatum (A, E, I, and J). Stroke strongly enhances the migration of newly born astrocytes (double-positive for tdTomato and GFAP, arrows in D and H; for single channel images, see Figure S4), but disallows the migration of DCX^+^ neuroblasts deeply into the lesion (B–D, I, and J). ISP treatment enhances DCX^+^ neuroblast migration (G, I, L, and N) but did not change total tdTomato^+^ cells (F, H, I, and J). ISP treatment enhanced the total number of DCX^+^ cells that penetrated into the glial scarred lesion area at 30 days post-stroke (K–O) and the migration of DCX^+^ into the glial scarred lesion area: (P)total DCX^+^ cell migrated area (Q) furthest distance migrated from the lateral wall of ventricle and (R) furthest distance migrated from the border of the glia scar in striatum. **p < 0.01 and ***p < 0.001, ANOVA for (I and J) and Student’s t test for (O–R). Each data point represents the average of 1 animal (average for each animal is obtained by quantifying multiple brain sections expanding the stroke infarct volume). Data were combined from 2 independent cohorts of mice.

To examine whether our *in vitro* findings translate to a stroke model, we investigated whether post-stroke ISP treatment would enhance SVZ and striatal neuroblast numbers and the migration of neuroblasts into the infarct area. To examine whether ISP treatment could enhance proliferation, migration, differentiation, and survival of NSCs and their progeny, we used an NSC-specific inducible cell labeling system (Jin et al., 2015; Lagace et al., 2007; Li et al., 2010), using the Ai9 tdTomato reporter. The nestin creERT2-YFP mouse has been previously used by us and others to label newly born cells after stroke (Jin et al., 2017; Li et al., 2010) and has successfully labeled multilineage progeny from nestin^+^ NSCs post-stroke. However, the Ai9 tdTomato reporter line has not been tested in a stroke model. Two cohorts of mice were used to examine the generation of new neuroblasts and their migration from the SVZ with WT C57BL/6J mice or the nestin creERT2-tdTomato mice. To ensure the specific labeling of SVZ-derived NSCs but not reactive astrocytes, which also upregulate nestin after stroke, we treated the nestin creERT2-tdTomato mice with tamoxifen (TAM) for 5 days starting 22 days before stroke (Figure 3). The waiting period of 17 days after TAM treatment allowed the clearing of TAM from the brain and did not label any reactive astrocytes at 2 days post-stroke in the striatum while it maintained the labeling of SVZ NSCs (Figure S2). Using this mouse line and consistent with our previous observations (Jin et al., 2017), migrations of DCX^+^ and tdTomato^+^ cells penetrated into the lesioned striatum at 30 days after stroke (Figure 3). There was minimal migration of DCX or tdTomato^+^ cells in the striatum on the contralateral side (Figures 3A and 3E). Scrambled or ISP peptides were administered starting 1 day post-stroke and then daily for 30 days. At 30 days post-stroke, on the ischemic side of the brain, ISP treatment resulted in clearly increased numbers of DCX^+^ cells as well as their enhanced migration toward the stroke penumbra but had no effect on the contralateral side (Figures 3B, 3C, 3F, and 3G, quantification in 3I). This lack of a contralateral effect is consistent with the results that 30 days of ISP treatment in non-stroke mice does not change total DCX^+^ or tdTomato cells in the SVZ (Figure S3). Interestingly, total DCX^+^ cells that had migrated well into the reactive astroglial infarct area also increased with ISP treatment, suggesting enhanced migration deep into the glial scar surrounding the injured area in the brain (Figures 3K-3O). Indeed, not only did the total number of DCX^+^ cells increase in ISP-treated mice (Figures 3K-3O) but also the total area covered by the DCX^+^ cells (quantification in Figure 3P), the furthest distance migrated from the lateral ventricular wall (quantification in Figure 3Q), and the furthest distance migrated from the medial glial scar border (quantification in Figure 3R) significantly increased in ISP-treated mice.

Different from the nestin creERT2-R26YFP reporter mice in which 30% of the DCX^+^ were also yellow fluorescent protein positive (YFP^+^) (Jin et al., 2017; Li et al., 2010), our results using the nestin creERT2-tdTomato (Ai9) mice showed that the vast majority of the tdTomato cells that had migrated into the striatum from the SVZ were clearly differentiating as glia, since they were doubly labeled with GFAP (Figures 3D and 3H, arrows; single channel images are included in Figure S4). Interestingly, the average number of migrated tdTomato^+^ cells in the striatum did not differ between control and ISP-treated mice (Figure 3J). However, within the SVZ proper and closely adjacent to its lateral wall, many cells that did contain tdTomato were also DCX^+^ (Figures 3B and 3F). Thus, primarily, the migration of DCX^+^ neuroblasts is being inhibited by CSPG-PTPσ interactions in the vicinity of the scar. The lack of abundant tdTomato expression in migrated neuroblasts deeper into the infarct zone could be due to downregulated activity of the promoter driving the reporter (tdTomato), which could, in turn, downregulate the reporter allele specifically in differentiating/migrating neuroblasts.

To further identify whether the increased number of DCX^+^ cells in the ipsilateral side of the brain as well as those that migrated into the glial scar area were due to increased proliferation of NSCs, in a different cohort, we harvested stroke mice that were subjected to scrambled peptide or ISP treatment after 14 days, a time when NSC cell proliferation peaks in response to stroke (Arvidsson et al., 2002; Palma-Tortosa et al., 2017). As before (Jin et al., 2017), NSCs were labeled at 17 days pre-stroke by TAM, and control or ISP peptides were given daily starting at 1 day after stroke. Mice were harvested at 14 days post-stroke and DCX^+^, tdTomato^+^, and Ki67^+^ (proliferating cells) along the SVZ were quantified (Figure S5). There were no differences in the total number of Ki67^+^ cells along the SVZ at 14 days post-stroke and there were similar numbers of DCX^+^ and tdTomato^+^ cells within the same regions (Figure S5). This suggests that ISP treatment does not increase the proliferation of NSCs within the SVZ but, rather, the increased total DCX^+^ cells in ISP-treated mice at a later time point (day 30, Figure 3) may be the result of enhanced survival of DCX^+^ neuroblasts that migrate into the infarct area. Indeed, when we compared the day 14 and day 30 stroke brains, we observed further migration of tdTomato^+^ and DCX^+^ cells toward the infarct area at day 30 compared to day 14, with increased DCX^+^ cells navigating more deeply into the striatum in ISP-treated mice (Figure S6, white arrows in B, E, H, and K). In addition, we observed chain-like tdTomato^+^/DCX^+^ newly born neuroblasts migrating from the lateral wall of the SVZ toward the infarct area, which were associated with astrocytes and blood vessels (Figures S6M-S6R), consistent with previous reports on the SVZ origin of neuroblasts in the stroke striatum (Jin et al., 2003; Parent et al., 2002) and the migration pattern of SVZ-derived neuroblasts after stroke (Jin et al., 2003; Ohab et al., 2006).

### Inhibition of CSPG-PTPσ signaling by ISP increases axonal sprouting

**Figure 4.**
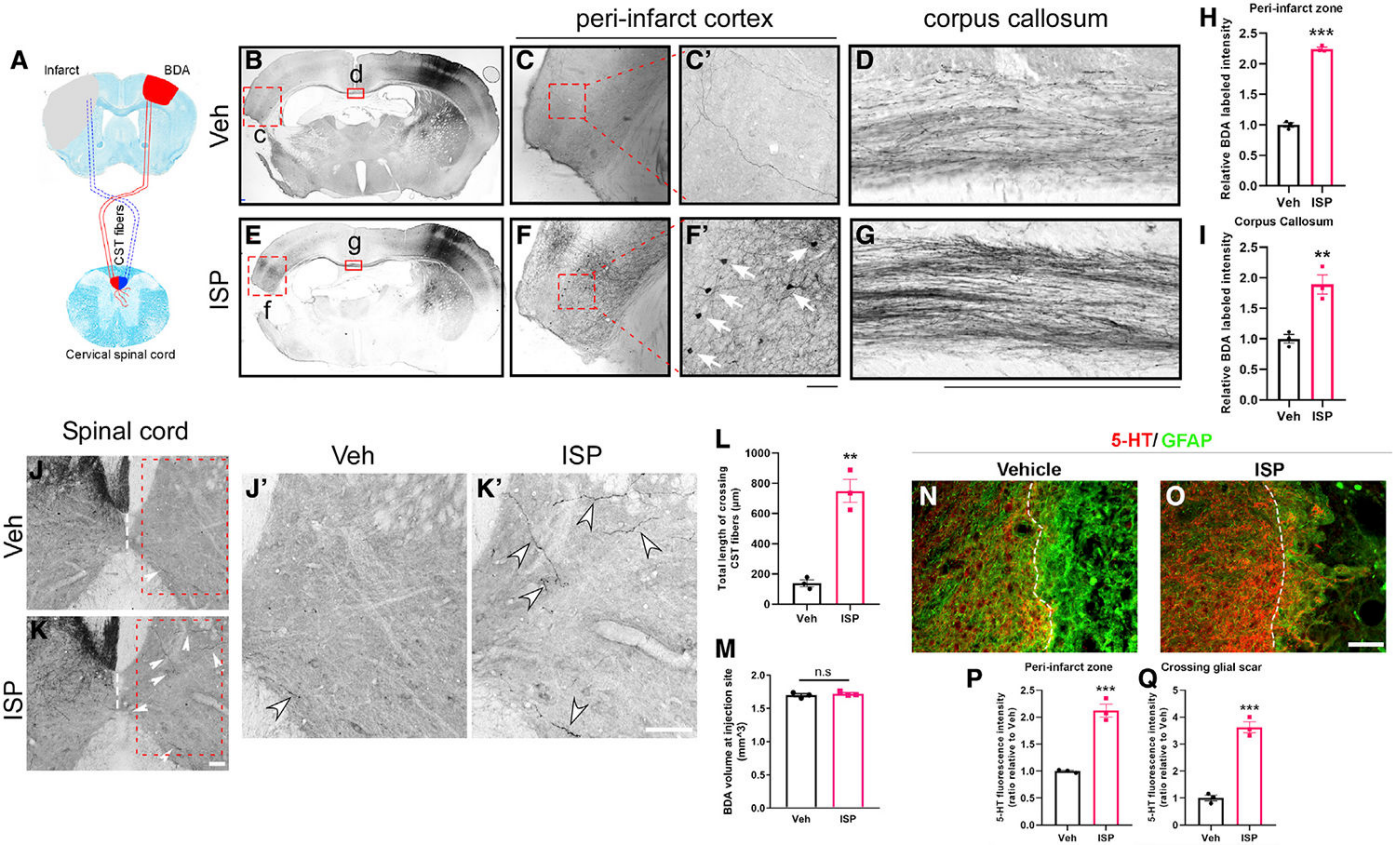
Post-stroke ISP treatment enhances axonal sprouting from the contralateral cortex after stroke (A) Schematic representation of the BDA injection site (contralateral to stroke side) and corticospinal tract (CST). (B–M) ISP enhances contralateral cross-callosal projections to the stroke side (B–D, vehicle (Veh)-treated stroke mice; E–G, ISP-treated stroke mice). Quantification in (H) and (I). ISP-treated mice show more callosal projecting neuronal cell bodies retrogradely labeled by BDA directly adjacent to the lesion. (C and F and higher magnification shown in C′ and F′). ISP also enhances the corticospinal tract (CST) sprouting from the non-stroke cortex to the contralateral side across the midline of the cervical spinal cord (J and K and J′ and K′ showing higher magnification). Quantification for CST cross-midline sprouting in (L). Note that BDA injection volume at the non-stroke cortex is similar in Veh or ISP-treated mice (representative images in B and E and quantification in M). (N and O) ISP treatment enhances the density of 5-HT^+^ axons that are in the peri-infarct area and crossing the glial scar region (quantification shown in P and Q). Scale bar: 100 μm (n = 3 mice for each group; multiple brain and spinal cord sections were analyzed for each mouse, and average was used as a single data point for statistical analysis). **p < 0.01 and ***p < 0.001, Student’s t test.

To examine whether post-stroke ISP treatment was also able to enhance axonal sprouting, another main mechanism of potential neurorepair after stroke, we examined axonal projections and sprouting from the contralateral motor-sensory cortex. The axonal tracer biotinylated dextran amine (BDA) (10,000 molecular weight [MW]) was injected in the contralateral intact primary motor-sensory cortex at 14 days post-stroke. Brains were harvested at 2 weeks after BDA injection and axonal projections to the peri-infarct area of the opposite hemisphere via the callosum were evaluated. Our results (Figures 4C, 4C′, 4F, and 4F′, quantification in 4H) revealed substantially increased axonal projections in the peri-infarct area in the stroke hemisphere originating from the contralateral cortex in ISP-treated mice. Consistent with this, labeled fiber intensity in the corpus callosum was increased by ISP treatment (Figures 4D and 4G, quantification in 4I). Interestingly, while this type of BDA tends to preferentially label anterogradely, in ISP-treated animals, we also observed some labeled neuronal cell bodies within the peri-infarct area in the stroke hemisphere but not in vehicle (Veh)-treated stroke mice (Figures 4C′ and 4F′), suggesting enhanced survival of peri-stroke neurons that normally project to the contralateral cortex. Naive non-stroke mice receiving BDA injection on one side of the cortex also showed similar retrogradely labeled neurons on the contralateral side in both Veh or ISP-treated mice following the same experimental timeline (Figure S7), suggesting that the preserved peri-stroke neurons in the ISP-treated group were not likely labeled due to the peptide somehow altering/enhancing retrograde uptake of BDA by cross-callosal neurons since they are also present in non-treated and treated naive mice. Focusing on the spinal cord, the recrossed contingent of BDA^+^ corticospinal axon terminals within the C3–C5 segments originating from the non-lesioned cortex was also significantly increased (Figures 4J, 4J′, 4K, and 4K′ with quantification in 4L). Injection volumes of BDA in the contralateral cortex were equal in Veh and ISP-treated stroke mice (Figure 4M).

Serotonergic fiber sprouting is well known to increase the tone and excitability of the injured CNS (Ghosh and Pearse, 2014). We, therefore, examined the 5-HT positive nerve terminals near the peri-infarct area at 4 weeks after stroke. Our data demonstrate that, in control animals, 5-HT fibers were present in the vicinity of the peri-infarct zone but were mainly stopped at the edge of the glial scar (Figure 4N). However, in ISP treated stroke mice, the intensity of 5-hydroxytryptamine-positive (5-HT^+^) fibers was increased within the peri-infarct zone and, in addition, serotonergic axons penetrated more deeply into the lesion epicenter, using the reactive scar astrocytes as their substrate (Figures 4O-4Q). We also examined the immunoreactive density of excitatory synaptic markers (presynaptic marker VGlut2 and postsynaptic marker Homer) at the peri-infarct cortex and found increased immunoreactivity of both VGlut2 and Homer in ISP-treated stroke mice (Figure S8), but no changes in ISP-treated naive mice (Figure S8), suggesting that post-stroke ISP treatment enhances the post-stroke peri-infarct zone synapse density without affecting the stability of synapses in naive non-stroke mice.

We also examined chronic atrophy (evaluated at 30 days post-stroke), which normally occurs within the lesioned side of the brain, in mice treated with either scrambled or ISP peptide started at either 1 day or 7 days after stroke. Compared to vehicle-treated stroke mice, ISP treatment started at either 1 day or 7 days post-stroke significantly decreased the extent of atrophy at 30 days after injury (Figure S9). The end of day 1 post-stroke-initiated treatment resulted in a more substantial decrease in brain atrophy compared to post-stroke treatment begun after 1 week. In addition, we noticed more lesion volume variation in day 7-treated mice compared to day 1-treated mice (Figure S9).

### ISP treatment alters the gene expression profile within the peri-stroke cortex

**Figure 5.**
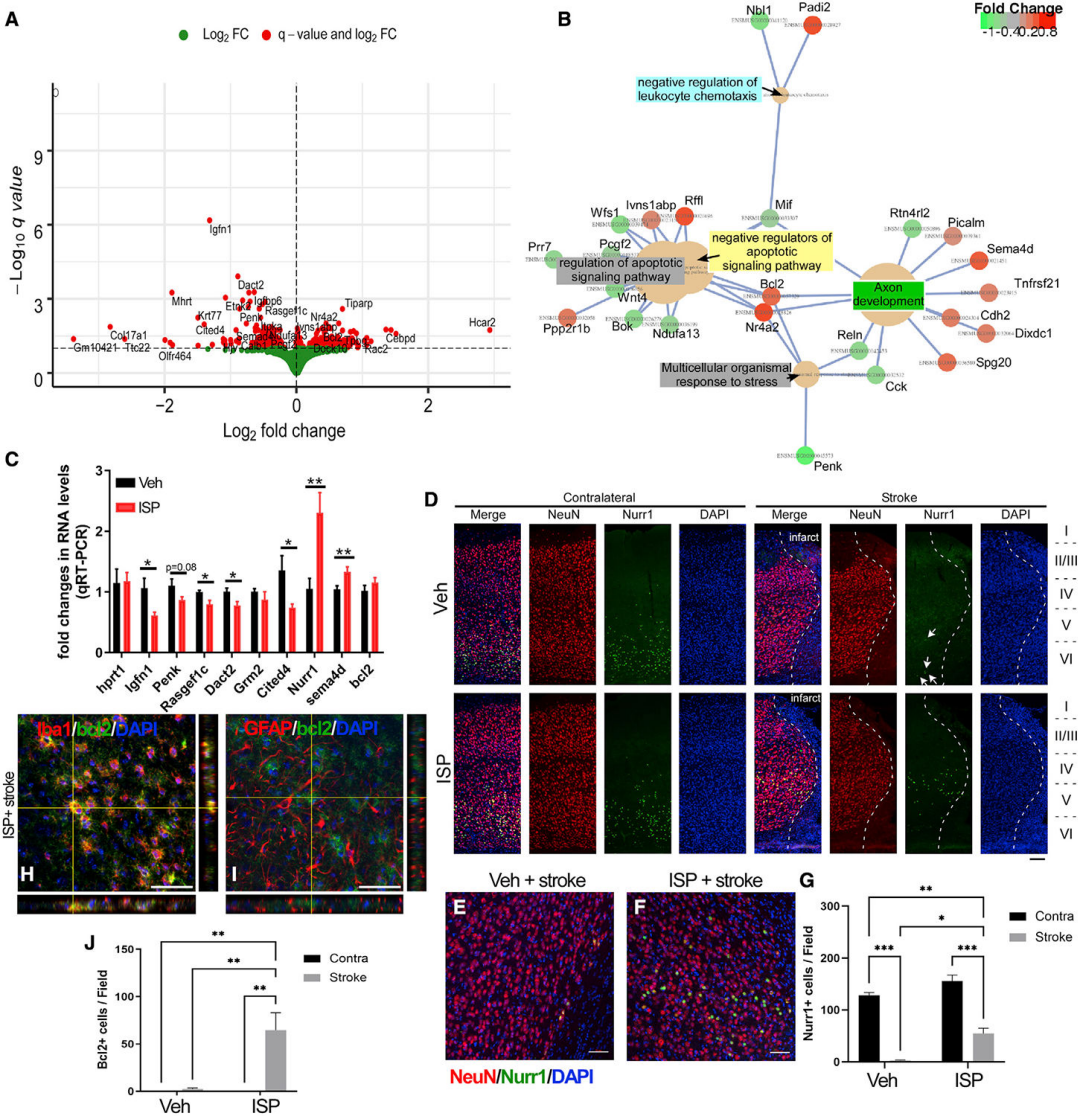
RNA-seq from the peri-infarct cortex of ISP treated versus Veh-treated stroke mice shows differentially regulated genes (a) DEGs that can be clustered into GO pathways (B) such as regulators of apoptotic signaling pathways, axon development pathways, and pathways that are involved in responses to stress. (C) Validation of the top selected gene expression by qRT-PCR (n = 7 for each group). (D–G) Nurr1 (Nr4a2) expression is decreased in peri-infarct cortex in stroke mice (arrows in D) but partially restored in ISP-treated mice (n = 4 for each group). Nurr1 expression is mainly detected in the NeuN^+^ neurons in the peri-infarct zone. (H–J) Bcl2 expression is upregulated in ISP-treated peri-infarct zone enriched in Iba1^+^ cells not GFAP^+^ reactive astrocytes (n = 4 for each group). *p < 0.05, **p < 0.01, ***p < 0.001, Student’s t test for (C) and 2-way ANOVA for (J) and (G). Scale bar=50 μm. See Table S2 for complete list of DEGs.

Given that ISP treatment improved axonal sprouting and the migration of new neuroblasts, preserved the integrity of peri-infarct-associated cortical neurons, and decreased overall atrophy of the stroke brain, we further explored the potential mechanism of ISP on promoting neuronal survival and function by examining the transcriptome changes in the peri-infarct cortical regions. We conducted RNA sequencing (RNA-seq) on tissue from motor cortex near the infarct zone on the stroke side from animals that received ISP or scrambled peptide starting at 1 day post-stroke. Cortical tissues were collected at day 14 post-stroke, a time point that has previously revealed transcriptomic differences in either behaviorally spontaneously recovered or non-recovered mice (Ito et al., 2018). RNA-seq showed that there were 217 genes upregulated and 185 genes downregulated within the motor cortex in ISP-treated mice (Figure 5A, complete list of differentially expressed genes are provided in Table S2). Gene Ontology (GO) term analysis suggests that the pathways that changed include multiple genes upregulated in the negative regulators of apoptotic pathways (*Rffl/Ivns1abp/Nr4a2/Bcl2*, Figure 5B) and downregulated genes in the positive regulators of apoptotic pathways (*Pcgf2/Bok/Mif/Prr7/Ndufa13/Wnt4/Wfs1*, Figure 5B). Interestingly, several genes related to axon development (*Sema4d/Tnfrsf21/Cdh2/Nr4a2/Dixdc1/Spg20/Picalm/Bcl2*, Figure 5B) were also enriched, which is consistent with the enhanced axonal sprouting in ISP-treated mice. Compared to the RNA-seq data comparing mice that showed spontaneous behavioral recovery to the ones that do not recover after stroke (Ito et al., 2018), we found three overlapping genes from our dataset that underwent similar changes in direction and extent of mRNA levels (*cited4*, *Sag,* and *Tpbg*). We examined the changes in mRNA levels by qRT-PCR in vehicle or ISP-treated peri-infarct cortex of the top 5 differentially expressed genes (*Igfn1*, *Penk, Rasgef1c, Dact2,* and *Grm2*), in addition to *cited4, Sag, Tpbg,* and genes implicated in cell survival and axon development (*Nr4a2,* also known as *Nurr1, Bcl2,* and *Sema4d*). A majority of the target genes were validated by qRT-PCR, with the exception of lowly abundant mRNAs (*Sag* and *Tpbg)* and *Bcl2* (Figure 5C). We tested commercially available antibodies for these genes and found that NR4A2 (NURR1) expression is downregulated in Veh-treated stroke mice in the peri-infarct cortex, but it is preserved in ISP-treated mice (Figures 5D-5G). NURR1^+^ cells were mainly NeuN^+^ neurons (Figures 5E and 5F). Interestingly, although Bcl2 expression changes were not validated by qRT-PCR, they were validated by immunostaining. B cell lymphoma 2 (BCL-2) immunostaining was substantially increased in the ISP-treated peri-infarct area and was mainly colocalized with Iba1^+^ microglia or infiltrating macrophages (Figures 5H-5J). The expression of BCL-2 in microglial cells is consistent with previous characterizations of BCL-2 expression patterns in the adult rodent brain (Merry et al., 1994; Sassone et al., 2013). We recently reported an effect of PTPσ inhibition on promoting a beneficial M2-like alternative neuroinflammatory response after SCI (Dyck et al., 2018). The upregulation of BCL-2 expression in microglia may facilitate this beneficial inflammatory profile post-stroke. The detailed mechanisms of these target genes warrant further investigation.

### Post-stroke ISP treatment (1 or 7 days after stroke) promotes functional recovery

**Figure 6.**
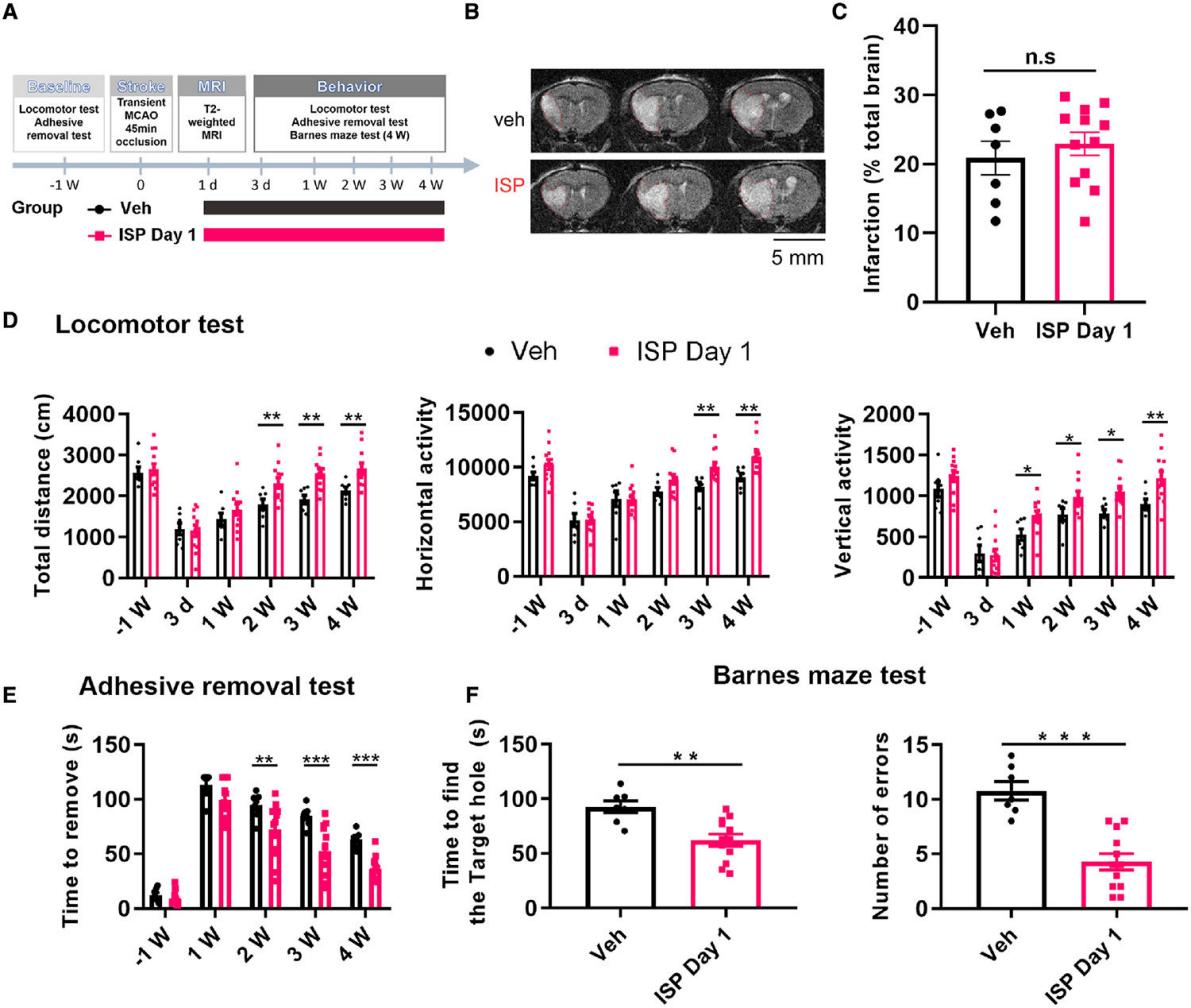
Post-stroke ISP treatment leads to enhanced functional recovery in mice (A) Experimental timeline. (B) Representative MRI images at different coronal levels. (C) At day 1 after stroke, before any treatment, the 2 groups of animals have similar infarct sizes and distributions. Post-stroke ISP treatment leads to enhanced general and fine locomotor functions as well as improved cognitive skills. (D) General locomotor performance was measured by automated open field chambers for 1 h. (E and F) Fine motor function was measured by the adhesive tape removal test (E) and cognitive function was measured by Barnes maze at 4 weeks after stroke (F). *p < 0.05, **p < 0.01, ***p < 0.001, 2-way repeated-measures (RM) ANOVA for (D) and (E) and Student’s t test for (F). Each data point represents an individual mouse, data pooled from 2 independent cohorts.

Given the positive effects mediated by ISP treatment in multiple neurorepair-related phenomena, we conducted a series of behavioral experiments to examine whether peptide treatment could enhance functional recovery in stroke animals. We tested the efficacy of continuous post-stroke ISP treatment (1 mg/kg/day, subcutaneously [s.c.], starting at 24 h post-stroke) using our proximal middle cerebral artery occlusion (MCAo) model. Two independent cohorts of young adult (10–12 weeks old) C57bl/6J male mice (total of n = 20 each group) were subjected to transient proximal MCAo surgery (45 min) to induce a large stroke in both striatal and cortical tissue (Figures 6A and 6B), mimicking a human “malignant” stroke (Carmichael, 2005). Stroke mice were subjected to T2-weighted MRI scanning to determine the size of the lesion epicenter and were comingled blindly into two equally distributed groups that either received daily vehicle or ISP treatment starting from 24 h post-stroke onset for 4 weeks. Just before the treatment was started, T2-weighted MRI showed that the two groups of mice had no differences in the extent and location of ischemic injury (Figures 6B and 6C). Using computer-monitored automated open field analysis, we found that ISP treatment significantly increased locomotor function at 2–4 weeks after stroke in multiple parameters (Figure 6D). Since the most common functional deficits following MCAo stroke are motor impairments of the contralateral upper limb, we also examined the effect of post-stroke ISP treatment on performance in a fine forelimb sensory-motor function test, “adhesive tape removal.” The results showed that post-stroke ISP treatment significantly improved the speed (Figure 6E) that mice were able to remove the tape from the contralateral affected paw (right front paw in our model) without any obvious effects on the time to remove the tape from the ipsilateral unaffected paw (left front paw in our model [Figure S10], showing the removal time on the unaffected left paw), suggesting that the result of ISP treatment is specifically related to stroke-induced deficits without untoward side effects in sensory and motor function. Importantly, at the 1-week pre-stroke time point and at post-stroke day 3, the control and the ISP-treated groups showed no differences in behavioral tests (post-stroke days 3 and 7 for open field and post-stroke day 7 for tape removal; Figures 6D and 6E), validating the equal grouping of animals according to stroke lesion size and supporting a neurorestorative mechanism through ISP treatment.

**Figure 7.**
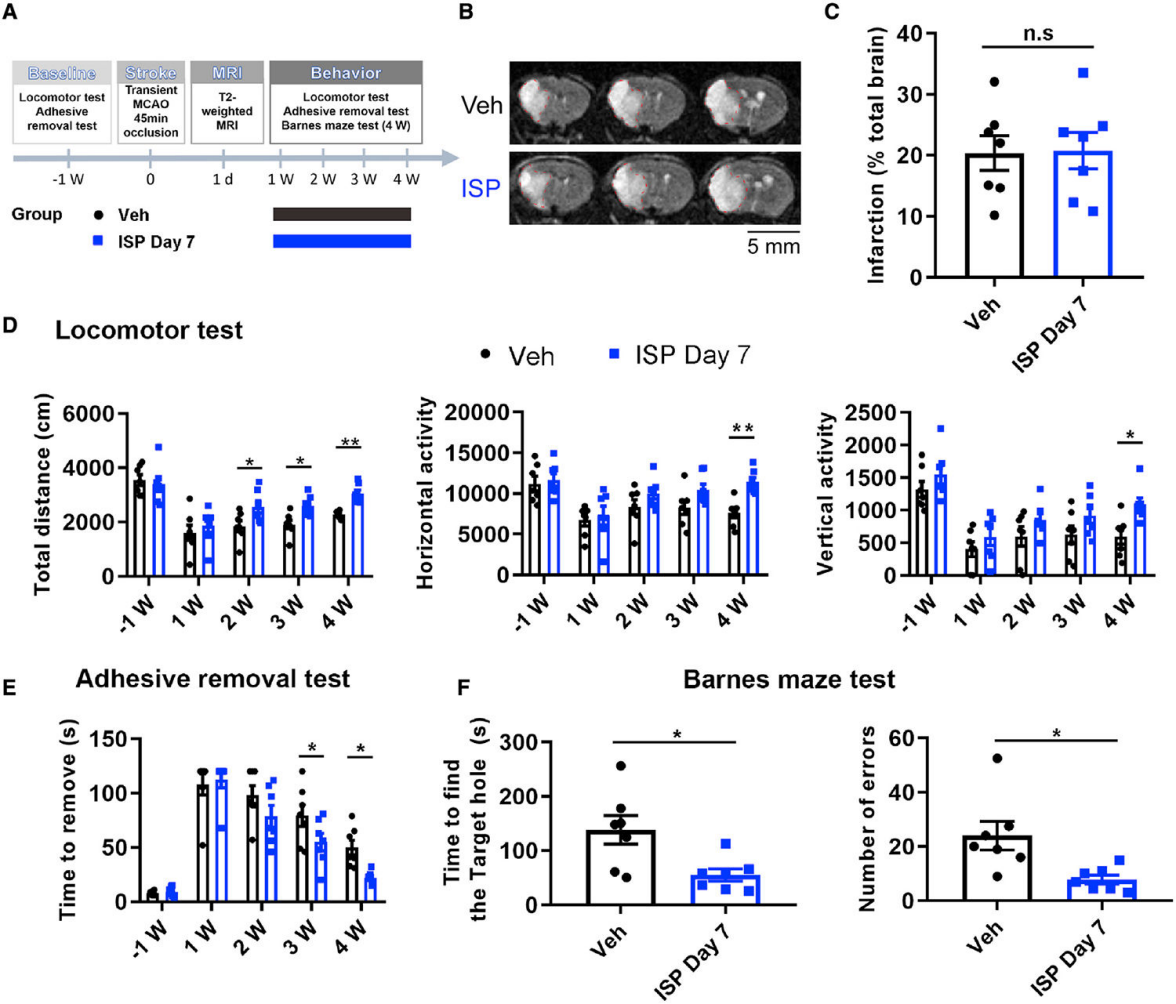
Post-stroke ISP treatment starting at post-stroke day 7 also leads to enhanced behavioral functional recovery in mice (A) Experimental timeline. (B) Representative MRI images at different coronal levels. (C) At day 1 after stroke, before any treatment, the 2 groups of animals have similar infarct sizes and distributions. (D–F) Post-stroke ISP treatment leads to enhanced general locomotor function measured by automated open field chambers for 1 h (D), increased fine motor function measured by the adhesive tape removal test (E), and improved cognitive function measured by Barnes maze at 4 w after stroke (F). *p < 0.05, **p < 0.01. Two-way RM ANOVA for (D) and (E) and Student’s t test for (F). Each data point represents an individual mouse; data from 1 cohort.

Since cognitive decline is also a major cause of disability in stroke survivors, we also examined the effect of ISP treatment on cognitive function. The Barnes maze test was used to evaluate learning/memory function, and our data showed that ISP-treated mice used significantly less time as well as fewer error trials to find the escape hole at 4 weeks post-stroke (Figure 6F). This demonstrates that systemic ISP treatment is able to improve multiple aspects of functional recovery, including general locomotor function, specific upper limb fine motor control, as well as cognitive function. To examine the potential effective time window of post-stroke ISP treatment, in a separate group of animals, we tested the efficacy of post-stroke treatment with ISP starting at day 7 after stroke. Our data (Figure 7) show that even when started 1 week after stroke, ISP treatment still effectively improved general locomotor function, specific upper limb sensorimotor function, and cognitive function. Note that in the locomotor function test, and especially in the fine motor tape removal test, the day that ISP-treated animals started to show significant improvements after delayed administration was equally shifted by approximately 1 week (Figure 7). However, the extent of functional recovery was only slightly reduced with delayed peptide administration, suggesting that the potential for neural recovery in stroke animals is still possible with a delayed onset of delivery of our regenerative peptide. Notably, for all three behavioral tests, significant improvements in ISP-treated mice in either post-day 1 or post-day 7 treatment paradigms all reached large effect sizes based on Cohen’s d and coefficient r (Cohen, 1988), especially toward more chronic time points (3–4 weeks post-stroke; statistical details on effect size provided in Tables S3 and S4), suggesting that the observed functional improvements due to ISP treatment are also biologically meaningful. Interestingly, when tested in non-stroke mice, ISP treatment in general did not result in significantly increased locomotor function as was observed in treated stroke mice (Figure S11A). At 3 weeks, for the total horizontal movement, the naive ISP group did show a slightly decreased range, which returned to the vehicle group level at 4 weeks. In the adhesive and cognitive functional tests (Figures S11B-S11D), there were no significant differences between the Veh-treated or ISP-treated naive groups.

## DISCUSSION

PTPσ, along with its sister phosphatase, leukocyte common antigen-related (LAR) have been identified as receptors for the inhibitory actions of CSPGs (Dickendesher et al., 2012; Fisher et al., 2011; Shen et al., 2009). The Lar family of receptors upregulates on neurons as well as certain types of NPCs after injury (Cregg et al., 2014; Dickendesher et al., 2012; Fisher et al., 2011; Gardner and Habecker, 2013; Kirkham et al., 2006; Lang et al., 2015; Shen et al., 2009). Upon lengthy interactions with CSPGs, PTPσ converts newly formed growth cones at the tips of severed axons or the leading processes of moving cells into a highly adhered and entrapped state (Lang et al., 2015). Thus, the biological effect of ISP, which specifically interferes with the function of PTPσ (Lang et al., 2015), could be far ranging and potentially efficacious in stroke by promoting regeneration/ sprouting or enhancing cell migrations over wide regions. Previous work by our group (Jin et al., 2017; Luo et al., 2009, 2013) and others (Jin et al., 2002; Zhang et al., 2001, 2006) has demonstrated that modulating endogenous neurogenesis is also a promising therapeutic paradigm for the treatment of stroke. While CSPGs have again been suggested to constrain the migrations of a variety of cell types, including oligodendrocyte progenitor cells (Lau et al., 2012), the role of CSPGs on repopulating NSCs in stroke remained unclear.

In this study, using a sizable CNS injury model (MCAo), we examined the role of the CSPG-PTPσ signaling pathway not only on axonal sprouting but also on injury-stimulated new neuroblast generation and migration, a phenomenon that has been much less explored. We have shown that post-stroke treatment with ISP improves behavioral recovery even in the delayed phase. Moreover, ISP treatment increased the total number of newly generated neuroblasts migrating well into the peri-infarct area. The peptide also enhanced serotonergic, callosal, and corticospinal tract (CST) regeneration/sprouting while increasing the density of excitatory synaptic markers at the peri-infarct zone. There was also significantly decreased cortical and striatal atrophy. Therefore, alleviating CSPG-mediated inhibition of a variety of neurorepair mechanisms is likely to be the molecular and cellular event that is allowing for enhanced recovery after stroke. Also, ISP may promote recovery from stroke by yet other mechanisms in addition to neuroprotection, neuroblast migration, or neuronal sprouting. It is known that members of the LAR family are important regulators of synaptic plasticity and neuronal physiology (Horn et al., 2012; Sclip and Sudhof, 2020), and they are important in the process of remyelination (Luo et al., 2018) phenomena that were not evaluated in this study.

Delayed secondary neuronal death contributes to the global tissue atrophy that occurs post-stroke (Sayed et al., 2020). Interestingly, we observed less atrophy in ISP-treated stroke animals, which was reflected at the cellular level by the survival of callosal neurons as well as NURR1^+^ neurons in the vicinity of the lesion penumbra. Multiple potential mechanisms could contribute to the diminished atrophy, including decreased delayed neuronal death, enhanced axonal remodeling, and altered neuroinflammation. Recent evidence has suggested that the chronic modulation of CSPG signaling via chondroitinase (Bartus et al., 2014; Didangelos et al., 2014) or the administration of our LAR family receptor blocking peptides in models of compressive SCI drives an anti-inflammatory and potently neuroprotective immune response (Dyck et al., 2018). It is likely that a similar pro-regenerative, ISP-induced immune phenotype may develop during ischemia brought on by stroke. The reduction in secondary damage also correlates well with the transcriptome profiling of the ISP-treated mice, such as enrichment of genes that negatively regulate apoptotic pathways as well as genes that are involved in axonal development. Interestingly, a recent study showed that a small pharmacological inhibitor of PTPσ also led to the upregulation of an anti-apoptotic gene BCL-X_L_ in hematopoietic stem cells (Zhang et al., 2019), suggesting a shared anti-apoptotic mechanism across different cell types. Our data suggest that BCL-2 protein expression is upregulated in Iba1^+^ microglia/macrophage cells near the peri-infarct zone, which agrees with previous reports of CNS BCL-2 expression patterns in adult brains (Merry et al., 1994; Sassone et al., 2013) and further supports that ISP may have a modulatory role in neuroinflammation after injury. Interestingly, our data showed that the same 30-day treatment of ISP in non-stroke mice did not affect the synaptic density in the cortex, suggesting that ISP treatment likely does not affect synaptic stability in the non-stroke brain. It is known that stroke injury stimulates a transcriptomic program in cortical neurons that facilitates synaptic reorganizations and plasticity (Joy and Carmichael, 2021), and this could explain why the effects of ISPs on synaptic density and sprouting of axons are more evident after injury.

It has been well established that CSPGs are enriched in NSCs and that they regulate neural stem cell proliferation and differentiation (Faissner and Reinhard, 2015; Galindo et al., 2018; Gates et al., 1995; Sirko et al., 2007; Yamada et al., 2018). However, the role of PTPσ on adult neural stem cells *in vivo* is not known, especially after a large injury such as an MCAo stroke. Our study provides evidence that post-stroke treatment with a PTPσ selective inhibitor enhanced the total number and migration of DCX^+^ neuroblasts well into the glial-scarred infarct area. DCX^+^ neuroblasts have been shown to contribute to functional recovery after stroke by ablation studies, despite the low numbers that can survive as maturing neurons (Jin et al., 2010; Sun et al., 2012), suggesting a mechanism beyond neuronal circuitry replacement. Newly born neuroblasts that migrate into the infarct zone could contribute to improved functional recovery by releasing neurotrophic factors that protect existing neuronal circuits or enhance tissue repair (Butti et al., 2012; Cuartero et al., 2021). An especially intriguing observation was that tdTomato^+^ stem cells, that appeared to differentiate toward an astroglial fate could migrate out of the SVZ and well into the lesion core laden with CSPGs, and their numbers did not increase in treated animals. However, DCX + neuron precursors were blocked at the edge of the scar only in the controls. It has been known that the infarct area in stroke upregulates several chemokines such as stromal-derived factor 1α (SDF-1α) (Ohab et al., 2006; Robin et al., 2006) and monocyte chemoattractant protein −1 (MCP-1) (Yan et al., 2007) that can attract the migration of neuroblasts through their expression of corresponding receptors CXR4 and CCR2. Meanwhile, differentiating neurons may upregulate the CSPG receptor more extensively or rapidly than their precursors. Thus, the mechanisms that differentially allow for lengthy migrations of stem cells but not neurons within a purportedly inhibitory environment likely depend on the balance of inhibitory versus growth-stimulating ECMs as well as the dynamics of the specific receptors that the cells produce as they encounter a variety of different terrains.

Another strategy that migrating cells or extending axons use to invade inhibitory regions is to produce matrix-degrading enzymes. MMPs have been implicated to guide neuroblast migration post-stroke (Grade et al., 2013; Lee et al., 2006). We recently reported a downstream pathway in OPCs regulated by PTPσ that involves specific CSPG-degrading enzymes (Tran et al., 2018a). Thus, ISP treatment upregulated the release of MMP2 in OPCs, which allows them to digest their way into CSPG-filled plaques, enhancing their remyelination potential in models of MS (Luo et al., 2018). Interestingly, here, we also report the upregulation of *Mmp2* RNAs in adult NSCs by ISP treatment, and the stimulatory effect of ISP on NSC migration is reversed by an MMP2 inhibitor. We have also documented that genetic deletion or ISP blockade of PTPσ in adult DRGs leads to the secretion of cathepsin B, which allows them to degrade and cross a strongly inhibitory gradient of CSPG (Tran et al., 2018a). Thus, a conserved signaling pathway in a variety of cell types appears to exist that links PTPσ to very particular matrix-digesting mechanisms that we have shown can be amplified experimentally to enhance the ability of cells or axonal growth cones to navigate within an inhibitory environment.

In summary, our data using both pharmacological and genetic PTPσ inhibition confirms and expands the inhibitory role of CSPGs in axonal plasticity and, in addition, demonstrates a critical role of the CSPG-PTPσ signaling cascade in the regulation of adult neural stem cell migration into regions undergoing scar formation and proteoglycan deposition. Such biological reparations may have implications in both normal physiological function and the regenerative response of the brain after injury.

### Limitations of the study

Our study has revealed multiple potential mechanisms that may mediate the beneficial regenerative effects of modulating the CSPG receptor PTPσ in post-stroke recovery. It is also possible that there are combined effects of neuroprotection and neural repair. It is difficult to rule out either or to determine whether one particular mechanism is a “primary” cause of functional recovery. At early time points post-stroke (day 3 after MCAo), there were no differences in behavioral deficits in Veh or ISP-treated mice (functional improvements become apparent at later time points), which suggests that recovery could at least be partly due to neural repair. Surely, the robust sprouting or regeneration of serotonergic axons into the lesion penumbra and deep into the lesion core along with spouting of the callosal and corticospinal systems could also be possible repair mechanisms in addition to neuroprotection. Our study was not able to determine whether there is a “primary” mechanism of the beneficial effects of ISP treatment in stroke mice. In addition, our study mainly focused on the generation, survival, and migration of neuroblasts, not new mature functional neurons. Previous studies have suggested that newly born neuroblasts may be critical for functional recovery after stroke and, therefore, their contribution may not fully depend on full maturation into functional neurons after stroke.

## STAR★METHODS

### RESOURCE AVAILABILITY

#### Lead contact

Further information and requests for resources and reagents should be directed to and will be fulfilled by the lead contact, Yu Luo (luoy2@ucmail.uc.edu).

#### Materials availability

This study did not generate new unique reagents.

#### Data and code availability

- RNA-seq data have been deposited at GEO and are publicly available as of the date of publication. Accession numbers are listed in the key resources table. Microscopy data and behavioral test data reported in this paper will be shared by the lead contact upon request.
- No original code was generated in this study.
- Any additional information required to reanalyze the data reported in this paper is available from the lead contact upon request.

### EXPERIMENTAL MODEL AND SUBJECT DETAILS

#### Animals

All animal protocols were approved by the IACUC of University of Cincinnati. C57BL/6J male mice were purchased from Jackson Laboratory and housed in the animal facility of the University of Cincinnati. Mice were maintained with a 12-hour light/dark cycle and fed ad libitum. To evaluate neurogenesis in stroke mice, both female and malenestin-CreERT2-Ai9 (inducible-tdTomato) strain was used in this study. The Nestin-CreERT2 mouse line was previously reported by Lagace et al. (Lagace et al., 2007) and was crossed with the Ai9 line (Madisen et al., 2010) to generate cre-inducible tdTomato expression in nestin+ neural stem cells (NSCs) and their progeny. No animals were excluded from data analysis. To generate *Ptprs* KO neural stem cells (NSCs), we obtained the *Ptrps*<tm1a(KOMP)Mbp (RRID:MGI:5797751) mouse line from KOMP (Bunin et al., 2015; Kim et al., 2020). The *Ptprs* loxP/loxP mouse line was generated by crossing C57BL/6N-Tg(CAG-Flpo)1Afst/Mmucd mouse with *Ptrps*<tm1a (KOMP)Mbp mice to get the conditional floxed *Ptprs* allele (Figure S1). The resulting mice were then outcrossed to remove the FLP allele. In this *Ptprs* loxP/loxP mouse line, exon 4 (3rd Open reading frame-containing exon) is deleted in the presence of cre recombinase, leading to an open reading frame shift that results in a pre-mature stop codon at the 96th aa. The recombined allele generates a 96 aa protein that is approximately 10KD instead of ~200KD of the wildtype protein (1805aa), thus leading to loss of function of the *Ptrps* gene. The loss of PTPσ protein from this *Ptrps* floxed allele upon cre expression has previously been validated (Kim et al., 2020).

#### Tamoxifen treatment *in vivo*

Nestin-CreERT2-R26R-Ai9 tdTomato mice (8–10 weeks old) were given tamoxifen dissolved in 10% EtOH/90% sunflower oil by gavage feeding at a dose of 180 mg/kg daily for 5 consecutive days. This dosing regimen was previously demonstrated to provide maximal recombination with minimal mortality and successfully monitored the activated SVZ NSCs stimulated by stroke (Jin et al., 2015; Lagace et al., 2007; Li et al., 2010). For NSC fate mapping, TAM treated mice received MCAo surgery 17 days after the last TAM administration and mice were perfused at 30 days post stroke to harvest brain for immunostaining. The time frame was chosen to specifically label NSCs in adult mice before the introduction of MCAo but also allow the clearance of TAM from mice at the time of MCAo to prevent labeling of reactive astrocytes which also upregulate nestin expression after stroke (Jin et al., 2015). To ensure this specific labeling we also examined whether the reactive astrocytes are labeled by reporter gene expression at 2 days post-stroke (Figure S2). To examine the effect of ISP in non-stroke mice, Tamoxifen was administered in the same timeline with the exception that mice were not subjected to MCAo surgery.

### METHOD DETAILS

#### Peptides synthesis

Peptides were purchased from CS-Bio (CA, USA) with >98% purity. Lyophilized peptides were dissolved in sterile water and stored at −80°C until use. Peptide sequences are as follows:

ISP: GRKKRRQRRRCDMAEHMERLKANDSLKLSQEYESI

Scrambled ISP (SISP): GRKKRRQRRRCIREDDSLMLYALAQEKKESNMHES

#### Neural stem cell culture

Primary neural stem cells were obtained from C57BL/6J mice at 5–6 weeks of age and neurosphere cultures were established as described previously (Reynolds and Weiss, 1992). After euthanization, whole brains were immediately harvested and dissected under the microscope to obtain the subventricular zone (SVZ) tissue. After mechanical dissociation with a knife, the tissue fragments were processed using trypsin and resuspended as individual cells at a density of 10^4^ cell/cm^2^ in neurobasal media and B27 with growth factors (epidermal growth factor and basic fibroblast growth factor). Subsequent passaging of cells was performed using Accutase® (innovative #AT-104, CA, USA) every 7 days until the cells established viable lines, and cellular debris was naturally diminished after each passage. At day 4 of each passage, the proliferating spheres were fed with media containing fresh growth factors. We used neurospheres at passage P3-P8 in this study. For *Ptrps* cKO NSCs, to avoid potential *in vivo* compensatory effects of *Ptrps* gene deletion, we prepared SVZ NSCs from *Ptrps* loxP/loxP mice (5–6 weeks old) (Bunin et al., 2015; Kim et al., 2020), and infected *Ptrps* loxP/loxP NSCs with AAV-CMV-Cre (Addgene) to delete *Ptrps* one passage before the differentiation and migration assay. Deletion of the floxed exon was confirmed by PCR (Figure S1).

#### Identification of secreted CSPGs from conditioned media of SVZ neurospheres

To measure secreted CSPGs by neurospheres, control or conditioned media were collected after SVZ spheres had been in culture for 3–4 days and subjected to concentration and mass spectrum (MS) analysis. Protocol (Table S1): The media (about 2mL) was filtered through a 100kDa filter (88523) by centrifuging at 4000 × g for 15 min according to the manufacturer’s instructions. The retentate was about 40ul. 10ul (25%) was mixed with 30ul of Invitrogen LDS sample buffer and run on a 4–12% Bis-Tris gel using MOPS buffer. The gel was silver stained. The sections chosen (roughly above 70K) were excised, destained, reduced with DTT and alkylated with IAA and digested with trypsin overnight. The resulting peptides were extracted and dried by speed vac. They were then resuspended in 7 ul of 0.1% Formic acid (FA). 5.5 uL of each sample was analyzed by nanoLC-MS/MS (Orbitrap Eclipse) and was searched against the *mus musculus* (uniprot 10090) and all entries database using Proteome discoverer ver 2.4 with the Sequest HT search algorithm. (Thermo scientific). Some representative CSPGs were shown in Figure 1 and the complete list of identified proteins is included in the Table S1. The only protein detected in control growth media was BSA which is a component of the B27 supplement.

#### Neural stem cell differentiation assay

Briefly, glass coverslips were coated with poly-ornithine and laminin. After dissociating neurospheres during passaging, individual cells were plated at a density of 1 × 10^4^ cells/cm^2^ in 500μL of growth media (Neurobasal media+ growth factors). Every other day, 250μL of media was removed from each well and 250μL of fresh NBM-GF was added. When the attached cells reached approximately 70% confluency (around day 5), all NBM-GF within each well was gently removed and immediately replaced with neurobasal media without growth factors (NBM) with ISP peptide (2.5μM) or scrambled peptide (2.5μM). Each well was fed daily by removing 250μL of the media and adding 250μL of the media containing ISP or scrambled peptide without growth factors. On Day 5 after complete replacement of the NBM-GF, the wells were fixed with 4% paraformaldehyde for 15 min at room temperature and stored in phosphate-buffered saline at 4°C until staining. Neuronally differentiated cells were identified by immunostaining using MAP2 or β-III tubulin and neurite length was quantified. Three coverslips were analyzed per condition. Random selections of fields in each coverslip were chosen and imaged by Stereo Investigator Software (MBF Bioscience, Williston, VT, USA), and a total of 50 cells (from 3 coverslips) were quantified by using NIH ImageJ software. Each experiment was repeated twice with similar results and representative results are presented.

#### Migration of neural stem cells on CSPGs

To determine the effects of CSPGs on the migration of neural stem cells *in vitro*, flat-bottom 48-well plates were coated with poly-L-lysine and various concentrations (1ug/mL and 10ug/mL) of Aggrecan (A1960, sigma). The control wells contained poly-L-lysine alone. Neurospheres of similar size were seeded in each well in NBM-GF medium with 2.5μM ISP peptide or scrambled peptide (n = 7 neurospheres per condition), and the plates were incubated at 37°C for 21 hours. Thereafter, images of each well were taken using a Leica DMi8 widefield microscope. The Migration Index was defined as dividing the total area of migrated cells by the inner area of neurospheres. The inner area and total area of neurospheres were measured using ImageJ software. Time lapse analyses (duration 21 hours) of the migration process (Video S1, Supplementary data) was taken using a Leica DMi8 widefield microscope equipped with an on-stage incubator. For application of signaling pathway inhibitors, neurospheres were treated with LY294002 (10μM, AKT inhibition), PD98059 (10μM, ERK inhibition) or OA-Hy (100nM, MMP2 inhibition) for 30min followed by exposure to 2.5μM ISP peptide or scrambled peptide. For ChABC treatment, neurospheres were incubated with 5 mU/mL ChABC (Sigma, # C2905) for 21 hours and then NSCs migration was measured. Each experiment was repeated twice with similar results and representative results are presented.

#### Western blot analysis

About 10 neurospheres were plated in a 60mm dish and subjected to ISP or scrambled peptide for 21 hours. NSCs were homogenized with RIPA lysis buffer and protein concentration was determined by Pierce BCA protein assay kit according to the manufacturer’s instructions (#23227, Thermo Fisher). Then, equal amounts of protein were loaded onto 15% or 12% SDS-PAGE gels, and electrophoretically transferred to PVDF membranes (Millipore). The membranes were blocked in 0.1% TPBS buffer with 5% BSA for 1 h at room temperature and incubated with indicated primary antibodies overnight at 4°C and followed by secondary antibodies conjugated to horseradish peroxidase. The following primary antibodies were used: AKT (#60203-2-Ig, Proteintech), ERK1/2 (#16443-1-AP, Proteintech), p-Akt (Ser473) (# 4060S, Cell signaling), GAPDH (#60004-1-Ig, Proteintech), p-ERK1/2 (Thr202/Tyr204) (# 4370S, Cell signaling). Enhanced chemiluminescence was performed with a West Pico Kit (Thermo Fisher). The density of bands was quantified using ImageJ software (NIH). This experiment was repeated three times and each pair of protein samples (scrambled peptide or ISP) were used for Western blot quantification.

#### Murine model of transient focal ischemia

Transient middle cerebral artery occlusion (tMCAo) was induced in male C57BL/6J or Nestin-creER-Ai9 mice (10–12 weeks old, 25–30g) by intraluminal occlusion of the left MCA for 45 min with a silicone rubber-coated monofilament (Cat.602212PK10Re and 602312PK10Re, Doccol Corporation). Briefly, mice were anesthetized with isoflurane. Body temperature was monitored and maintained at 37 ± 0.5°C by a homeothermic blanket control unit (Harvard apparatus). To minimize pain, mice were subcutaneously injected with buprenorphine. A midline incision was made in the skin overlying the calvarium and the skin was pulled laterally to fix a flexible microtip on the surface of the left parietal bone of the skull of the mice (0.5 mm posterior and 3.5 mm lateral to the bregma). Next, a midline neck incision was made to isolate the left common carotid artery (CCA), external carotid artery (ECA), and internal carotid artery (ICA). A silicone rubber-coated monofilament was introduced via the arteriotomy in ECA and advanced slowly through ICA toward the origin of the MCA according to Longa’s method (Longa et al., 1989). To ensure consistent and successful blockage of the MCA, regional cerebral blood flow was monitored in all stroke animals by Laser Doppler flowmetry (PeriFlux system 5000, Perimed, Sweden). After incision closure, mice were subcutaneously given 1mL warm saline and placed in a heated animal intensive care unit until recovery. T2-weighted MRI imaging at 18 hours post stroke before any treatment was given to ensure equal grouping.

#### Magnetic resonance imaging (MRI)

Infarct volumes were measured using a horizontal biospec 9.4T scanner with a 3-cm birdcage coil (Bruker Inc., Billerica, MA)23h after induction of brain ischemia. During MRI scanning procedures, mice were anesthetized with a 1.5% isoflurane/oxygen mixture and placed in the cradle in a prone position. The body temperature of the mouse was maintained at 33°C by blowing warm air into the scanner through a feedback control system (SA Instruments, Stony Brook, NY). The respiration rate was also monitored during the experiments. To quantify ischemic edema volume, multi-slice, T2-weighted, axial images were acquired using a rapid acquisition with relaxation enhancement (RARE) sequence with the following parameters: TE/TR, 15/2000 ms; RARE factor, 8; NAV, 4; matrix size, 256 × 256; slice thickness, 1mm; number of slices, 13; field of view, 2.4 × 2.4cm. Image reconstruction and analyses were performed using in-house developed, MATLAB-based software (Natick, MA, USA). ROIs of ischemic edema volume and brain tissue were drawn manually from T2-weighted images. Consequently, the percentage of ischemic infarct volume was calculated as following formula to correct for edema in infarct: Σ (contralateral area – ipsilateral non-infarct area)/Σcontralateral area X100% as described previously (Lin et al., 1993).

#### Systemic peptide treatment

After MRI scanning, ischemic mice were divided into two equally distributed groups according to the size of the stroke injury and the two groups were randomly assigned as either control or treatment cohort by flipping a coin. At 24h or 7 d post ischemia and each afternoon thereafter until 4 weeks after stroke, mice were subcutaneously injected under the skin of the lower back with ISP (1μg/g/day) or vehicle (same volume of 5% DMSO in saline). Subsequent analyses were carried out in a blinded manner and the treatment groups were revealed after the data analysis.

#### Quantification of brain atrophy in stroke animals using Giemsa staining

4 weeks post stroke, brains were harvested and subjected to Giemsa staining as described previously (Turcato et al., 2018). Briefly, post-stroke 4-week brain sections (25 μm) were mounted on PLL-coated slides. The sections were rehydrated in KH2PO4 buffer (pH 4.5) for 10 min, and then stained in pre-warmed 10% Giemsa solution for 30 min at 42°C. After a brief rinse with KH2PO4 buffer, sections were dehydrated in absolute ethanol, cleared in xylene and mounted with Histoseal. A set of serial sections was imaged by a Path Scan Enabler IV slide scanner. Contralateral and ipsilateral brain areas were quantified using ImageJ software. The calculation formula of atrophy rate is as follows: Σ (contralateral brain area – ipsilateral brain area)/Σcontralateral brain area X100%.

#### Anterograde tracing and quantification of axonal sprouting

Two weeks after tMCAo, mice were injected with 1.5 μL (0.5ul per site) of the biotin dextran amine (BDA, MW10,000; 10% in PBS, Invitrogen) at three sites in the contralesional cortex (coordinates: 1. A/P 0.0 mm, M/L −2mm, D/V −1mm; 2.A/P 0.5mm, M/L −1.5mm, D/V −1mm; 3. A/P 0.5mm, M/L −2mm, D/V −1mm). Two weeks after injection (4 weeks after stroke), the brain and cervical spinal cord were harvested after cardiac perfusion with PBS followed by 4% paraformaldehyde. An additional cohort of naïve non-stroke mice were subjected to daily Veh or ISP treatment received BDA injection following the same timeline. After post-fixation overnight in 4% paraformaldehyde and cryoprotection in 20% and 30% sucrose, coronal brain sections and transverse spinal cord sections were cut at 30 μm thickness. For the detection of BDA, sections were rinsed in 0.1M PB and incubated in 0.3% H2O2 for 30 min to inactivate endogenous peroxidase, followed by incubation for 2 hours with a Vectastain ® ABC kit (Vector Laboratories, Burlingame, CA, USA). Staining was developed with 2,3′ diaminobenziine tetrahydrochloride (0.5mg/mL in 0.1M PB). The numbers and lengths of spinal cord midline-crossing BDA + fibers and callosal fibers were assessed by an experimenter that was blinded to the treatment information. Sections were analyzed with ImageJ software. At least three sections at similar coronal levels in the immediate vicinity of the peri-infarct cortex (between AP 0.0–0.5mm) and at spinal cord segments (C3-C5) were chosen for each animal. All immunohistochemical measurements were done by blinded observers.

#### Immunohistochemistry

Mice were anesthetized and perfused with PBS and 4% paraformaldehyde (PFA). The brain was dissected and post-fixed in 4% PFA overnight at 4°C and equilibrated in 20% and 30% sucrose. 30 μm-thick sections were blocked in 4% BSA/0.3% Triton-x100 for 1 hour. After blocking, sections were incubated with primary antibodies overnight at 4°C and followed by appropriate secondary antibodies conjugated with Alexa fluorescence 488, 555 or 647. For Nurr1 staining, tissue sections were incubated with citrate buffer at 80°C for 30 minutes and followed by normal immunostaining protocols. The following primary antibodies were used in this study: 5-HT (1:500, Immunostar, Hudson, WI), CS56 (1:500, C8035, Sigma), DCX (1:1000, Cell Signaling), Ki67 (1:200, Invitrogen), GFAP (1:1000 Sigma), NURR1 (1:100 R&D systems), VGlut2 (1:1000 Millipore), Homer1 (1:1000 Millipore), BCL-2 (1:100 Santa Cruz) and IBA1 (1:1000 Wako). Omission of primary or secondary antibodies resulted in no staining and served as negative controls. Group and treatment information was all blinded to the image analyzer. Images were acquired by a motorized stage-equipped Leica DM5000B microscope (Leica Microsystems, Bannockburn, IL) and a Zeiss LSM710 LIVE Duo confocal microscope. Unbiased quantification was performed with Stereo Investigator image software (MBF Bioscience, Williston, VT). Quantification of cell proliferation and number of newly born neuroblasts was carried out as described in our previous publication (Jin et al., 2017; Luo et al., 2020). In brief, SVZ region Ki67 or DCX+ or tdTomato positive cells were quantified on both the ipsilateral and contralateral sides. For quantification of newly-born neuroblasts and migration of SVZ NSC-derived cells into the infarct area, total tdTomato+ or DCX+ cells were quantified in the GFAP+ astrocyte containing area. For quantification of synaptic puncta, immunoreactivity of VGlut2 or Homer1 positive puncta at 3 adjacent locations within the peri-infraction cortex region and corresponding area in non-stroke mice on each section for 3 sections per animal were measured with ImageJ software. For quantification of NURR1 positive cells, NURR1 positive cells within the peri-infraction cortex area and corresponding area on the contralateral side on each section for 3 sections per animal were counted by ImageJ software. At least 3 sections containing the region of interest at the similar coronal location were quantified for all studies and values (total or average) for each animal were considered as one data point for statistical analysis. Omission of primary or secondary antibodies resulted in no staining and served as negative controls. Group and treatment information was all blinded to the image analyzer.

#### RNAseq analysis of peri-infarct cortical tissue and qRT-PCR validation in Veh and ISP treated stroke mice

Stroke mice received either vehicle or ISP peptide (n = 4 for each group) starting from post stroke day 1 (psd1) and daily for 14 days. At 14 days post stroke, mouse brains were quickly extracted and snap frozen. The frozen tissues were stored at −80°C until further processing. Peri-infarct cortex (2mm range: sensorimotor cortex and motor cortex) were micro-punched using a Harris Micro-punch 1 mm in diameter and 1 mm in depth. Total RNA was isolated using an RNeasy micro kit (Qiagen, Germany). The extracted RNA that passes the quality control by Novogene was processed for cDNA library preparation. The cDNA product was amplified, and sequencing adapters and barcodes were ligated onto the fragments for each sample to create cDNA libraries ready for sequencing. Library preparation and sequencing was performed by Novogene Corporation Inc. (USA) using state-of-the-art Illumina NovaSeq platform. Downstream analysis was performed using a combination of programs including Hisat2, HTseq, and R package DESeq2. Alignments were parsed using Hisat2 program and mapped to mouse reference genome (mm10) (Kim et al., 2019). Mouse genes annotation was obtained from GENCODE. Read counts matrix for each gene in each sample was calculated by HTseq program with default parameters and differentially expressed were determined through DESeq2 (Anders et al., 2015; Love et al., 2014). GO and KEGG enrichment were implemented by the g:Profiler and ClusterProfiler (Raudvere et al., 2019). Gene fusion and differences in alternative splicing events were detected by Star-fusion and rMATS software. Differential Expression Analysis Results were performed by using the DESeq2 R package, while the significant criterion is padj <0.1 and log2 fold change >0.5. Complete sequencing results have been uploaded to the NCBI Gene Expression Omnibus (GEO) repository and can be downloaded with accession number GSE168934. RNA expression levels were validated in an independent cohort of mice (n = 7 for each group) at post-stroke day 14 at the same peri-infarct cortical location. Tissues and RNA were extracted using the same method described above. Total RNA (1 ug) was treated with RQ-1 Rnase-free Dnase I and reverse transcribed into cDNA using random hexamers by Superscript III reverse transcriptase (Life Sciences). cDNA levels for HPRT1 (hypoxanthine phosphoribosyltransferase 1), Hmbs (hydroxymethylbilane synthase) and various target genes were determined, using specific primer/probe sets by quantitative RT-PCR using a Roche Light Cycler II 480. Relative expression level was calculated using the delta Ct method compared to Hmbs as a reference gene. Primers and carboxyfluorescein (FAM) labeled probes used in the quantitative RT-PCR for each gene are listed in the oligonucleotide section.

#### Neurobehavioral assays

All behavioral tests were performed during the light phase in a blinded fashion. To reduce stress, mice were acclimated in the behavioral test room 1h before beginning. All apparatuses were cleaned with 75% ethanol in between animals to avoid behavioral reactions to odorant differences between mice. Locomotor function tests were carried out at pre (during one week before stroke) and 3, 7, 14, 21, 28 days after tMCAo as previously described (Jin et al., 2017; Luo et al., 2009). Barnes Maze test was carried out at 28 days post stroke as described previously (Jin et al., 2017). Adhesive removal test was performed on days 7, 14, 21 and 28 post-stroke as described previously (Luo et al., 2020). To achieve an optimum level of performance, mice were trained for 4 days in the week before stroke surgery to establish the baseline for the adhesive removal test. An additional cohort of naïve non-stroke mice received similar daily injection of Veh or ISP and are subjected to same time line for all behavioral tests. Details for all behavioral tests are listed below.

#### Locomotor function

Mice motor activities were assessed using automated open field Accuscan activity monitors (Columbus, OH, USA) in the week before and 3, 7, 14, 21, 28 days after tMCAo as previously described (Jin et al., 2017; Luo et al., 2009, 2020). There are 16 horizontal and 8 vertical infrared sensors (interval 2.5 cm) in each chamber. Each mouse was put into a 42 × 42 × 31 cm Plexiglas open box for 1 hour with food and water supply. To avoid observer bias, this locomotor test was automatically monitored by the computer and software. Locomotor activity was calculated by automated Fusion software (Accuscan, Columbus, OH, USA). The following variables were measured: (A) horizontal activity (the total number of beam interruptions that occurred in the horizontal sensors); (B) total distance traveled (cm, the distance traveled by the animals); (C) Vertical activity (the total number of beam interruptions that occurred in vertical sensors).

#### Adhesive removal test

This test was performed on days 7, 14, 21 and 28 post-stroke in order to examine fine motor deficits. Each mouse was placed into a transparent cylinder (15 cm diameter) during a habituation period of 1min. Thereafter, two different colored adhesive labels (2.5mm diameter made by punch, Tough Spots) were applied with equal pressure on each mouse’s forepaw. The time to remove the adhesive labels from each paw was measured with a maximum of 2 min. Time to remove the tape from each forepaw was quantified by an observer that is blinded to the treatment information. To achieve an optimum level of performance, mice were trained for 4 days before surgery.

#### Barnes maze test

The spatial memory of ischemic mice was examined using a Barnes maze (Stoelting company, WoodDale, IL, USA) 28 days after tMCAo as described previously (Jin et al., 2017). The maze consists of a 91.5 cm diameter circular platform with 20 holes (19 closed bottom, 1 open bottom with escape chamber) around the platform perimeter with various colored shapes attached to the walls around the testing arena. Mice were discouraged from being sedentary or to idle around aimlessly by the presence of noisy overhead blowing fans and a bright light above the platform. At day 0, mice were gently guided to enter the open bottom target hole after removing the start chamber. At day 1, mice were trained for 4 trials in 2 sessions to find the escape tunnel placed under the target hole. Once mice entered the target hole, the hole was covered and mice were allowed to stay in it for 2min. If mice could not locate the target hole within 5 min, mice were guided by the observer to enter the target hole. At day 2, one trial was run and video-taped until the mouse getting into the target hole or stopped at 5 mins when the mouse could not locate the target hole. Time spent to locate the escaping hole and error numbers in finding the hiding hole made by the mouse were measured by an observer blinded to animal treatment group.

### QUANTIFICATION AND STATISTICAL ANALYSIS

All studies were analyzed using SigmaPlot. Results are expressed by mean ± SEM of the indicated number of experiments. Statistical analysis was performed using the Student’s t test, and one- or two-way analysis of variance (ANOVA), as appropriate, with Tukey post hoc tests or Bonferroni post hoc tests for repeated behavioral measurements. A p value equal to or less than 0.05 was considered significant. Effect size on behavioral tests is calculated according to Cohen’s d and Coefficient r values as described (Cohen, 1988). Effect size for each test at each time point and p value from post-hoc analysis in RM Two-way ANOVA is listed in Tables S3 and S4.

## Supplementary Material

1

2

3

4

## Figures and Tables

**Table:** KEY RESOURCES TABLE

REAGENT or RESOURCE	SOURCE	IDENTIFIER
Antibodies		
Rabbit 5-HT (Serotonin) Antibody	ImmunoStar	Cat#20080, RRID: AB_572263
Mouse Monoclonal Anti-Chondroitin Sulfate antibody	Sigma-Aldrich	Cat#C8035, RRID: AB_476879
Rabbit Doublecortin (DCX) Antibody	Cell Signaling Technology	Cat#4604, RRID:AB_561007
Mouse Monoclonal Anti-Glial Fibrillary Acidic Protein (GFAP) antibody	Sigma-Aldrich	Cat# G3893, RRID:AB_477010
Rat Ki-67 monoclonal antibody (SolA15)	Invitrogen	Cat#14-5698-82, RRID:AB_10854564
Goat Nurr1 Polyclonal antibody	R & D Systems	Car#AF2156, RRID:AB_2153894
Guinea pig VGluT2 polyclonal antibody	Sigma-Aldrich	Cat#AB2251-I, RRID:AB_2665454
Rabbit Homer1 polyclonal antibody	Sigma-Aldrich	Cat#ABN37, RRID:AB_11214387
Mouse Bcl-2 (C-2) antibody	Santa Cruz	Cat#sc-7382, RRID:AB_626736
Rabbit Iba1 antibody	FUJIFILM Wako	Cat#019-19741, RRID:AB_839504
Mouse AKT antibody	Proteintech	Cat#60203-2-lg, RRID:AB_10912803
Rabbit ERK1/2 antibody	Proteintech	Cat#16443-1-AP, RRID:AB_10603369
Rabbit p-Akt (Ser473) antibody	Cell Signaling Technology	Cat#4060S, RRID:AB_2315049
Mouse GAPDH antibody	Proteintech	Cat# 60004-1-Ig, RRID:AB_2107436
Rabbit p-ERK1/2 (Thr202/Tyr204) antibody	Cell Signaling Technology	Cat# 4370, RRID:AB_2315112
VECTASTAIN ABC-Peroxidase Kit	Vector Laboratories	Cat# PK-4001, RRID:AB_2336810
Chemicals, peptides, and recombinant proteins		
ISP	CS-Bio	N/A
Scrambled ISP (SISP)	CS-Bio	N/A
LY294002	Sigma-Aldrich	Cat# 19-142
PD98059	Sigma-Aldrich	Cat# P215
OA-Hy	Sigma-Aldrich	Cat# 444244
Chondroitinase ABC	Sigma-Aldrich	Cat# C2905
Aggrecan	Sigma-Aldrich	Cat# A1960
Biotin Dextran Amine	Invitrogen	Cat# D1956
Deposited data		
RNA-seq data	This paper	GEO:GES168934
Experimental models: Organisms/strains		
Mouse: Ptprstm1b(KOMP)Mbp/Ptprs+	KOMP	MGI Cat# 5797751, RRID:MGI:5797751
Mouse: C57BL/6-Tg(Nes-cre/ERT2)KEisc/J	The Jackson Laboratory	IMSR Cat# JAX:016261, RRID:IMSR_JAX:016261
Mouse: B6.Cg-Gt(ROSA)26Sortm9 (CAG-tdTomato)Hze/J	The Jackson Laboratory	IMSR Cat# JAX:007909, RRID:IMSR_JAX:007909
Mouse: B6N(B6J)-Tg(CAG-Flpo) 1Afst/Mmucd	MMRRC	MMRRC Cat# 036512-UCD, RRID:MMRRC_036512-UCD
Oligonucleotides		
Primers: Hprt1	IDT	F: TGA TAG ATC CAT TCC TAT GAC TGT AGA R: AAG ACA TTC TTT CCA GTT AAA GTT GAG
Primers: Igfn1	Applied Biosystems	Assay ID: Mm00617360_m1
Primers: Penk	IDT	F: AAC ACC GGC AAT GGA CTG R: AAA CTC GCC TGG ATT TTG G
Primers: Rasgef1c	Applied Biosystems	Assay ID: Mm01288803_m1
Primers: Dact2	Applied Biosystems	Assay ID: Mm00555888_m1
Primers: Grm2	Applied Biosystems	Assay ID: Mm01235831_m1
Primers: Cited4	IDT	F: CCG AGA ACA CCT GCC TTG R: AGC GAG ACC CAA CTG TCA TC
Primers: Nurr1	IDT	F: TCA GAG CCC ACG TCG ATT R: TAG TCA GGG TTT GCC TGG AA
Primers: Sema4d	IDT	F: AAG TGG GTG CGC TAC AAT G R: GGG CCT CAC TGT CGA TAC AC
Primers: Bcl2	IDT	F: GTA CCT GAA CCG GCA TCT G R: GGG GCC ATA TAG TTC CAC AA
Primers:MMP2	IDT	F: CAG GGA ATG AGT ACT GGG TCT ATT R: ACT CCA GTT AAA GGC AGC ATC TAC
Primers:GAPDH	IDT	F: TGC CAA ATA TGA TGA CAT CAA GAA R: GGA GTG GGT GTC GCT GTT G
Software and algorithms		
Stereo Investigator	MBF Bioscience	RRID:SCR_017667
ImageJ	NIH	RRID:SCR_003070
MATLAB	Mathworks	RRID:SCR_001622
SigmaPlot	SigmaPlot	RRID:SCR_003210

## Highlights

- Upregulation of CSPGs after stroke blocks neuroblast migration and axon sprouting
- RPTPσ modulating peptide (ISP) overcomes the inhibition and improves stroke recovery
- DCX + neuroblasts migrate deep into the scar region after ISP treatment
- Sprouting of several axon tracts is enhanced with relevant transcriptomic changes.

## References

[R1] Anders S, Pyl PT, and Huber W (2015). HTSeq–a Python framework to work with high-throughput sequencing data. Bioinformatics 31, 166–169. 10.1093/bioinformatics/btu638. 25260700 PMC4287950

[R2] Arvidsson A, Collin T, Kirik D, Kokaia Z, and Lindvall O (2002). Neuronal replacement from endogenous precursors in the adult brain after stroke. Nat. Med 8, 963–970. 12161747 10.1038/nm747

[R3] Bartus K, James ND, Didangelos A, Bosch KD, Verhaagen J, Yáñez-Muñoz RJ, Rogers JH, Schneider BL, Muir EM, and Bradbury EJ (2014). Large-scale chondroitin sulfate proteoglycan digestion with chondroitinase gene therapy leads to reduced pathology and modulates macrophage phenotype following spinal cord contusion injury. J. Neurosci 34, 4822–4836. 10.1523/JNEUROSCI.4369-13.2014. 24695702 PMC3972714

[R4] Benjamin EJ, Blaha MJ, Chiuve SE, Cushman M, Das SR, Deo R, de Ferranti SD, Floyd J, Fornage M, Gillespie C, (2017). Heart disease and stroke statistics-2017 update: a report from the American heart association. Circulation 135, e146–e603. 10.1161/CIR.0000000000000485. 28122885 PMC5408160

[R5] Bunin A, Sisirak V, Ghosh HS, Grajkowska LT, Hou ZE, Miron M, Yang C, Ceribelli M, Uetani N, Chaperot L, (2015). Protein tyrosine phosphatase PTPRS is an inhibitory receptor on human and murine plasmacytoid dendritic cells. Immunity 43, 277–288. 10.1016/j.immuni.2015.07.009. 26231120 PMC4547994

[R6] Burnside ER, De Winter F, Didangelos A, James ND, Andreica EC, Layard-Horsfall H, Muir EM, Verhaagen J, and Bradbury EJ (2018). Immune-evasive gene switch enables regulated delivery of chondroitinase after spinal cord injury. Brain 141, 2362–2381. 10.1093/brain/awy158. 29912283 PMC6061881

[R7] Butti E, Bacigaluppi M, Rossi S, Cambiaghi M, Bari M, Cebrian Silla A, Brambilla E, Musella A, De Ceglia R, Teneud L, (2012). Subventricular zone neural progenitors protect striatal neurons from glutamatergic excitotoxicity. Brain 135, 3320–3335. 10.1093/brain/aws194. 23008234

[R8] Carmichael ST (2005). Rodent models of focal stroke: size, mechanism, and purpose. NeuroRx 2, 396–409. 10.1602/neurorx.2.3.396. 16389304 PMC1144484

[R9] Carmichael ST (2008). Themes and strategies for studying the biology of stroke recovery in the poststroke epoch. Stroke 39, 1380–1388. 10.1161/STROKEAHA.107.499962. 18309162 PMC2711539

[R10] Carmichael ST (2010).Targets for neural repair therapies after stroke. Stroke 41, S124–S126. 10.1161/STROKEAHA.110.597146. 20876486 PMC2955885

[R11] Chen XR, Liao SJ, Ye LX, Gong Q, Ding Q, Zeng JS, and Yu J (2014). Neuroprotective effect of chondroitinase ABC on primary and secondary brain injury after stroke in hypertensive rats. Brain Res. 1543, 324–333. 10.1016/j.brainres.2013.12.002. 24326094

[R12] Chopp M, and Li Y (2008). Treatment of stroke and intracerebral hemorrhage with cellular and pharmacological restorative therapies. Acta Neurochir. Suppl 105, 79–83. 19066087 10.1007/978-3-211-09469-3_16

[R13] Cohen J (1988). Statistical Power Analysis for the Behavioral Sciences (New York, NY: Routledge Academic).

[R14] Cregg JM, DePaul MA, Filous AR, Lang BT, Tran A, and Silver J (2014). Functional regeneration beyond the glial scar. Exp. Neurol 253, 197–207. 10.1016/j.expneurol.2013.12.024. 24424280 PMC3951813

[R15] Cuartero MI, García-Culebras A, Torres-López C, Medina V, Fraga E, Vázquez-Reyes S, Jarerño-Flores T, García-Segura JM, Lizasoain I, and Moro MÁ (2021). Post-stroke neurogenesis: friend or foe? Front. Cell Dev. Biol 9, 657846. 10.3389/fcell.2021.657846. 33834025 PMC8021779

[R16] Dempsey RJ, Sailor KA, Bowen KK, Türeyen K, and Vemuganti R (2003). Stroke-induced progenitor cell proliferation in adult spontaneously hypertensive rat brain: effect of exogenous IGF-1 and GDNF. J. Neurochem 87, 586–597. 14535942 10.1046/j.1471-4159.2003.02022.x

[R17] Dickendesher TL, Baldwin KT, Mironova YA, Koriyama Y, Raiker SJ, Askew KL, Wood A, Geoffroy CG, Zheng B, Liepmann CD, (2012). NgR1 and NgR3 are receptors for chondroitin sulfate proteoglycans. Nat. Neurosci 15, 703–712. 10.1038/nn.3070. 22406547 PMC3337880

[R18] Didangelos A, Iberl M, Vinsland E, Bartus K, and Bradbury EJ (2014). Regulation of IL-10 by chondroitinase ABC promotes a distinct immune response following spinal cord injury. J. Neurosci 34, 16424–16432. 10.1523/JNEUROSCI.2927-14.2014. 25471580 PMC4252552

[R19] Dyck SM, and Karimi-Abdolrezaee S (2015). Chondroitin sulfate proteoglycans: key modulators in the developing and pathologic central nervous system. Exp. Neurol 269, 169–187. 10.1016/j.expneurol.2015.04.006. 25900055

[R20] Dyck SM, Alizadeh A, Santhosh KT, Proulx EH, Wu CL, and Karimi-Abdolrezaee S (2015). Chondroitin sulfate proteoglycans negatively modulate spinal cord neural precursor cells by signaling through LAR and RPTPsigma and modulation of the Rho/ROCK pathway. Stem Cell 33, 2550–2563. 10.1002/stem.1979. 25703008

[R21] Dyck S, Kataria H, Alizadeh A, Santhosh KT, Lang B, Silver J, and Karimi-Abdolrezaee S (2018). Perturbing chondroitin sulfate proteoglycan signaling through LAR and PTPsigma receptors promotes a beneficial inflammatory response following spinal cord injury. J. Neuroinflammation 15, 90. 10.1186/s12974-018-1128-2. 29558941 PMC5861616

[R22] Dyck S, Kataria H, Akbari-Kelachayeh K, Silver J, and Karimi-Abdolrezaee S (2019). LAR and PTPsigma receptors are negative regulators of oligodendrogenesis and oligodendrocyte integrity in spinal cord injury. Glia 67, 125–145. 10.1002/glia.23533. 30394599

[R23] Faissner A, and Reinhard J (2015). The extracellular matrix compartment of neural stem and glial progenitor cells. Glia 63, 1330–1349. 10.1002/glia.22839. 25913849

[R24] Filous AR, and Silver J (2016). Targeting astrocytes in CNS injury and disease: a translational research approach. Prog. Neurobiol 144, 173–187. 10.1016/j.pneurobio.2016.03.009. 27026202 PMC5035184

[R25] Fisher D, Xing B, Dill J, Li H, Hoang HH, Zhao Z, Yang XL, Bachoo R, Cannon S, Longo FM, (2011). Leukocyte common antigen-related phosphatase is a functional receptor for chondroitin sulfate proteoglycan axon growth inhibitors. J. Neurosci 31, 14051–14066. 10.1523/JNEUROSCI.1737-11.2011. 21976490 PMC3220601

[R26] Galindo LT, Mundim MTVV, Pinto AS, Chiarantin GMD, Almeida MES, Lamers ML, Horwitz AR, Santos MF, and Porcionatto M (2018). Chondroitin sulfate impairs neural stem cell migration through ROCK activation. Mol. Neurobiol 55, 3185–3195. 10.1007/s12035-017-0565-8. 28477140 PMC5842503

[R27] Gardner RT, and Habecker BA (2013). Infarct-derived chondroitin sulfate proteoglycans prevent sympathetic reinnervation after cardiac ischemia-reperfusion injury. J. Neurosci 33, 7175–7183. 10.1523/JNEUROSCI.5866-12.2013. 23616527 PMC3671889

[R28] Gates MA, Thomas LB, Howard EM, Laywell ED, Sajin B, Faissner A, Gotz B, Silver J, and Steindler DA (1995). Cell and molecular analysis of the developing and adult mouse subventricular zone of the cerebral hemispheres. J. Comp. Neurol 361, 249–266. 10.1002/cne.903610205. 8543661

[R29] Gherardini L, Gennaro M, and Pizzorusso T (2015). Perilesional treatment with chondroitinase ABC and motor training promote functional recovery after stroke in rats. Cereb. Cortex 25, 202–212. 10.1093/cercor/bht217. 23960208

[R30] Ghosh M, and Pearse DD (2014). The role of the serotonergic system in locomotor recovery after spinal cord injury. Front. Neural Circuits 8, 151. 10.3389/fncir.2014.00151. 25709569 PMC4321350

[R31] Gilman S (2006). Pharmacologic management of ischemic stroke: relevance to stem cell therapy. Exp. Neurol 199, 28–36. 16631744 10.1016/j.expneurol.2006.03.002

[R32] Goldstein LB (2007). Acute ischemic stroke treatment in 2007. Circulation 116, 1504–1514. 17893286 10.1161/CIRCULATIONAHA.106.670885

[R33] Grade S, Weng YC, Snapyan M, Kriz J, Malva JO, and Saghatelyan A (2013). Brain-derived neurotrophic factor promotes vasculature-associated migration of neuronal precursors toward the ischemic striatum. PLoS One 8, e55039. 10.1371/journal.pone.0055039. 23383048 PMC3558494

[R34] Hettiaratchi MH, O’Meara MJ, Teal CJ, Payne SL, Pickering AJ, and Shoichet MS (2019). Local delivery of stabilized chondroitinase ABC degrades chondroitin sulfate proteoglycans in stroke-injured rat brains. J. Control. Release 297,14–25. 10.1016/j.jconrel.2019.01.033. 30690102

[R35] Hettiaratchi MH, O’Meara MJ, O’Meara TR, Pickering AJ, Letko-Khait N, and Shoichet MS (2020). Reengineering biocatalysts: computational redesign of chondroitinase ABC improves efficacy and stability. Sci. Adv 6, eabc6378. 10.1126/sciadv.abc6378. 32875119 PMC7438101

[R36] Horn KE, Xu B, Gobert D, Hamam BN, Thompson KM, Wu CL, Bouchard JF, Uetani N, Racine RJ, Tremblay ML, (2012). Receptor protein tyrosine phosphatase sigma regulates synapse structure, function and plasticity. J. Neurochem 122, 147–161. 10.1111/j.1471-4159.2012.07762.x. 22519304

[R37] Huang L, Wu ZB, Zhuge Q, Zheng W, Shao B, Wang B, Sun F, and Jin K (2014). Glial scar formation occurs in the human brain after ischemic stroke. Int. J. Med. Sci 11, 344–348. 10.7150/ijms.8140. 24578611 PMC3936028

[R38] Ida M, Shuo T, Hirano K, Tokita Y, Nakanishi K, Matsui F, Aono S, Fujita H, Fujiwara Y, Kaji T, and Oohira A (2006). Identification and functions of chondroitin sulfate in the milieu of neural stem cells. J. Biol. Chem 281, 5982–5991. 10.1074/jbc.M507130200. 16373347

[R39] Ito M, Aswendt M, Lee AG, Ishizaka S, Cao Z, Wang EH, Levy SL, Smerin DL, McNab JA, Zeineh M, (2018). RNA-sequencing analysis revealed a distinct motor cortex transcriptome in spontaneously recovered mice after stroke. Stroke 49, 2191–2199. 10.1161/STROKEAHA.118.021508. 30354987 PMC6205731

[R40] Jin K, Zhu Y, Sun Y, Mao XO, Xie L, and Greenberg DA (2002). Vascular endothelial growth factor (VEGF) stimulates neurogenesis in vitro and in vivo. Proc. Natl. Acad. Sci. USA 99, 11946–11950. 12181492 10.1073/pnas.182296499 PMC129374

[R41] Jin K, Sun Y, Xie L, Peel A, Mao XO, Batteur S, and Greenberg DA (2003). Directed migration of neuronal precursors into the ischemic cerebral cortex and striatum. Mol. Cell. Neurosci 24, 171–189. 10.1016/s1044-7431(03)00159-3. 14550778

[R42] Jin K, Wang X, Xie L, Mao XO, and Greenberg DA (2010). Transgenic ablation of doublecortin-expressing cells suppresses adult neurogenesis and worsens stroke outcome in mice. Proc. Natl. Acad. Sci. USA 107, 7993–7998. 10.1073/pnas.1000154107. 20385829 PMC2867852

[R43] Jin Y, Raviv N, Barnett A, Bambakidis NC, Filichia E, and Luo Y (2015). The shh signaling pathway is upregulated in multiple cell types in cortical ischemia and influences the outcome of stroke in an animal model. PLoS One 10, e0124657. 10.1371/journal.pone.0124657. 25927436 PMC4415811

[R44] Jin Y, Barnett A, Zhang Y, Yu X, and Luo Y (2017). Poststroke sonic hedgehog agonist treatment improves functional recovery by enhancing neurogenesis and angiogenesis. Stroke 48, 1636–1645. 10.1161/STROKEAHA.117.016650. 28487338 PMC5667564

[R45] Joy MT, and Carmichael ST (2021). Encouraging an excitable brain state: mechanisms of brain repair in stroke. Nat. Rev. Neurosci 22, 38–53. 10.1038/s41583-020-00396-7. 33184469 PMC10625167

[R46] Kazanis I, and ffrench-Constant C (2011). Extracellular matrix and the neural stem cell niche. Dev. Neurobiol 71, 1006–1017. 10.1002/dneu.20970. 21898854 PMC4557210

[R47] Kim D, Paggi JM, Park C, Bennett C, and Salzberg SL (2019). Graph-based genome alignment and genotyping with HISAT2 and HISAT-genotype. Nat. Biotechnol 37, 907–915. 10.1038/s41587-019-0201-4. 31375807 PMC7605509

[R48] Kim K, Shin W, Kang M, Lee S, Kim D, Kang R, Jung Y, Cho Y, Yang E, Kim H, (2020). Presynaptic PTPsigma regulates postsynaptic NMDA receptor function through direct adhesion-independent mechanisms. Elife 9, e54224. 10.7554/eLife.54224. 32142410 PMC7069723

[R49] Kirkham DL, Pacey LKK, Axford MM, Siu R, Rotin D, and Doering LC (2006). Neural stem cells from protein tyrosine phosphatase sigma knockout mice generate an altered neuronal phenotype in culture. BMC Neurosci. 7, 50. 10.1186/1471-2202-7-50. 16784531 PMC1570144

[R50] Lagace DC, Whitman MC, Noonan MA, Ables JL, DeCarolis NA, Arguello AA, Donovan MH, Fischer SJ, Farnbauch LA, Beech RD, (2007). Dynamic contribution of nestin-expressing stem cells to adult neurogenesis. J. Neurosci 27, 12623–12629. 10.1523/JNEURO-SCI.3812-07.2007. 18003841 PMC3718551

[R51] Lang BT, Cregg JM, DePaul MA, Tran AP, Xu K, Dyck SM, Madalena KM, Brown BP, Weng YL, Li S, (2015). Modulation of the proteoglycan receptor PTPsigma promotes recovery after spinal cord injury. Nature 518, 404–408. 10.1038/nature13974. 25470046 PMC4336236

[R52] Lau LW, Keough MB, Haylock-Jacobs S, Cua R, Döring A, Sloka S, Stirling DP, Rivest S, and Yong VW (2012). Chondroitin sulfate proteoglycans in demyelinated lesions impair remyelination. Ann. Neurol 72, 419–432. 10.1002/ana.23599. 23034914

[R53] Lee SR, Kim HY, Rogowska J, Zhao BQ, Bhide P, Parent JM, and Lo EH (2006). Involvement of matrix metalloproteinase in neuroblast cell migration from the subventricular zone after stroke. J. Neurosci 26, 3491–3495. 10.1523/JNEUROSCI.4085-05.2006. 16571756 PMC6673870

[R54] Lee H, McKeon RJ, and Bellamkonda RV (2010). Sustained delivery of thermostabilized chABC enhances axonal sprouting and functional recovery after spinal cord injury. Proc. Natl. Acad. Sci. USA 107, 3340–3345. 10.1073/pnas.0905437106. 19884507 PMC2840440

[R55] Li L, Harms KM, Ventura PB, Lagace DC, Eisch AJ, and Cunningham LA (2010). Focal cerebral ischemia induces a multilineage cytogenic response from adult subventricular zone that is predominantly gliogenic. Glia 58, 1610–1619. 10.1002/glia.21033. 20578055 PMC2919586

[R56] Lin TN, He YY, Wu G, Khan M, and Hsu CY (1993). Effect of brain edema on infarct volume in a focal cerebral ischemia model in rats. Stroke 24, 117–121. 10.1161/01.str.24.1.117. 8418534

[R57] Longa EZ, Weinstein PR, Carlson S, and Cummins R (1989). Reversible middle cerebral artery occlusion without craniectomy in rats. Stroke 20, 84–91. 10.1161/01.str.20.1.84. 2643202

[R58] Love MI, Huber W, and Anders S (2014). Moderated estimation of fold change and dispersion for RNA-seq data with DESeq2. Genome Biol. 15, 550. 10.1186/s13059-014-0550-8. 25516281 PMC4302049

[R59] Luo Y, Kuo CC, Shen H, Chou J, Greig NH, Hoffer BJ, and Wang Y (2009). Delayed treatment with a p53 inhibitor enhances recovery in stroke brain. Ann. Neurol 65, 520–530. 10.1002/ana.21592. 19475672 PMC2690614

[R60] Luo Y, Shen H, Liu HS, Yu SJ, Reiner DJ, Harvey BK, Hoffer BJ, Yang Y, and Wang Y (2013). CART peptide induces neuroregeneration in stroke rats. J. Cereb. Blood Flow Metab 33, 300–310. 10.1038/jcbfm.2012.172. 23211962 PMC3564201

[R61] Luo F, Tran AP, Xin L, Sanapala C, Lang BT, Silver J, and Yang Y (2018). Modulation of proteoglycan receptor PTPsigma enhances MMP-2 activity to promote recovery from multiple sclerosis. Nat. Commun 9, 4126. 10.1038/s41467-018-06505-6. 30297691 PMC6175851

[R62] Luo F, Zhang Z, Barnett A, Bellinger TJ, Turcato F, Schmidt K, and Luo Y (2020). Cuprizone-induced demyelination under physiological and post-stroke condition leads to decreased neurogenesis response in adult mouse brain. Exp. Neurol 326, 113168. 10.1016/j.expneurol.2019.113168. 31904386 PMC9694109

[R63] Madisen L, Zwingman TA, Sunkin SM, Oh SW, Zariwala HA, Gu H, Ng LL, Palmiter RD, Hawrylycz MJ, Jones AR, (2010). A robust and high-throughput Cre reporting and characterization system for the whole mouse brain. Nat. Neurosci 13, 133–140. 10.1038/nn.2467. 20023653 PMC2840225

[R64] McKeon RJ, Schreiber RC, Rudge JS, and Silver J (1991). Reduction of neurite outgrowth in a model of glial scarring following CNS injury is correlated with the expression of inhibitory molecules on reactive astrocytes. J. Neurosci 11, 3398–3411. 1719160 10.1523/JNEUROSCI.11-11-03398.1991 PMC6575543

[R65] Merry DE, Veis DJ, Hickey WF, and Korsmeyer SJ (1994). bcl-2 protein expression is widespread in the developing nervous system and retained in the adult PNS. Development 120, 301–311. 8149910 10.1242/dev.120.2.301

[R66] Ohab JJ, Fleming S, Blesch A, and Carmichael ST (2006). A neurovascular niche for neurogenesis after stroke. J. Neurosci 26, 13007–13016. 10.1523/JNEUROSCI.4323-06.2006. 17167090 PMC6674957

[R67] Ohtake Y, Wong D, Abdul-Muneer PM, Selzer ME, and Li S (2016). Two PTP receptors mediate CSPG inhibition by convergent and divergent signaling pathways in neurons. Sci. Rep 6, 37152. 10.1038/srep37152. 27849007 PMC5111048

[R68] Okuda H (2018). A review of functional heterogeneity among astrocytes and the CS56-specific antibody-mediated detection of a subpopulation of astrocytes in adult brains. Anat. Sci. Int 93, 161–168. 10.1007/s12565-017-0420-z. 29086253

[R69] Palma-Tortosa S, García-Culebras A, Moraga A, Hurtado O, Perez-Ruiz A, Durán-Laforet V, Parra J.d.l., Cuartero MI, Pradillo JM, Moro MA, and Lizasoain I (2017). Specific features of SVZ neurogenesis after cortical ischemia: a longitudinal study. Sci. Rep 7, 16343. 10.1038/s41598-017-16109-7. 29180821 PMC5703956

[R70] Parent JM, Vexler ZS, Gong C, Derugin N, and Ferriero DM (2002). Rat forebrain neurogenesis and striatal neuron replacement after focal stroke. Ann. Neurol 52, 802–813. 10.1002/ana.10393. 12447935

[R71] Raudvere U, Kolberg L, Kuzmin I, Arak T, Adler P, Peterson H, and Vilo J (2019). g:Profiler: a web server for functional enrichment analysis and conversions of gene lists. Nucleic Acids Res. 47, W191–W198. 10.1093/nar/gkz369. 31066453 PMC6602461

[R72] Reynolds BA, and Weiss S (1992). Generation of neurons and astrocytes from isolated cells of the adult mammalian central nervous system. Science 255, 1707–1710. 1553558 10.1126/science.1553558

[R73] Rink S, Arnold D, Wöhler A, Bendella H, Meyer C, Manthou M, Papamitsou T, Sarikcioglu L, and Angelov DN (2018). Recovery after spinal cord injury by modulation of the proteoglycan receptor PTPsigma. Exp. Neurol. 309, 148–159. 10.1016/j.expneurol.2018.08.003. 30118740

[R74] Robin AM, Zhang ZG, Wang L, Zhang RL, Katakowski M, Zhang L, Wang Y, Zhang C, and Chopp M (2006). Stromal cell-derived factor 1alpha mediates neural progenitor cell motility after focal cerebral ischemia. J. Cereb. Blood Flow Metab 26, 125–134. 10.1038/sj.jcbfm.9600172. 15959456

[R75] Sakamoto K, Ozaki T, Ko YC, Tsai CF, Gong Y, Morozumi M, Ishikawa Y, Uchimura K, Nadanaka S, Kitagawa H, (2019). Glycan sulfation patterns define autophagy flux at axon tip via PTPRsigma-cortactin axis. Nat. Chem. Biol 15, 699–709. 10.1038/s41589-019-0274-x. 31061498

[R76] Sassone J, Maraschi A, Sassone F, Silani V, and Ciammola A (2013). Defining the role of the Bcl-2 family proteins in Huntington’s disease. Cell Death Dis. 4, e772. 10.1038/cddis.2013.300. 23949221 PMC3763461

[R77] Sayed MA, Eldahshan W, Abdelbary M, Pillai B, Althomali W, Johnson MH, Arbab AS, Ergul A, and Fagan SC (2020). Stroke promotes the development of brain atrophy and delayed cell death in hypertensive rats. Sci. Rep 10, 20233. 10.1038/s41598-020-75450-6. 33214598 PMC7678843

[R78] Sclip A, and Sudhof TC (2020). LAR receptor phospho-tyrosine phosphatases regulate NMDA-receptor responses. Elife 9, e53406. 10.7554/eLife.53406. 31985401 PMC6984820

[R79] Shen Y, Tenney AP, Busch SA, Horn KP, Cuascut FX, Liu K, He Z, Silver J, and Flanagan JG (2009). PTPsigma is a receptor for chondroitin sulfate proteoglycan, an inhibitor of neural regeneration. Science 326, 592–596. 10.1126/science.1178310. 19833921 PMC2811318

[R80] Siebert JR, and Osterhout DJ (2021). Select neurotrophins promote oligodendrocyte progenitor cell process outgrowth in the presence of chondroitin sulfate proteoglycans. J. Neurosci. Res 99, 1009–1023. 10.1002/jnr.24780. 33453083 PMC7986866

[R81] Silver J, and Miller JH (2004). Regeneration beyond the glial scar. Nat. Rev. Neurosci 5, 146–156. 10.1038/nrn1326. 14735117

[R82] Sirko S, von Holst A, Wizenmann A, Götz M, and Faissner A (2007). Chondroitin sulfate glycosaminoglycans control proliferation, radial glia cell differentiation and neurogenesis in neural stem/progenitor cells. Development 134, 2727–2738. 10.1242/dev.02871. 17596283

[R83] Sirko S, Akita K, Von Holst A, and Faissner A (2010). Structural and functional analysis of chondroitin sulfate proteoglycans in the neural stem cell niche. Methods Enzymol. 479, 37–71. 10.1016/S0076-6879(10)79003-0. 20816159

[R84] Soleman S, Yip PK, Duricki DA, and Moon LDF (2012). Delayed treatment with chondroitinase ABC promotes sensorimotor recovery and plasticity after stroke in aged rats. Brain 135, 1210–1223. 10.1093/brain/aws027. 22396394 PMC5581954

[R85] Sun F, Wang X, Mao X, Xie L, and Jin K (2012). Ablation of neurogenesis attenuates recovery of motor function after focal cerebral ischemia in middle-aged mice. PLoSOne 7, e46326. 10.1371/journal.pone.0046326. PMC3482223 23110048

[R86] Tran AP, Sundar S, Yu M, Lang BT, and Silver J (2018a). Modulation of receptor protein tyrosine phosphatase sigma increases chondroitin sulfate proteoglycan degradation through cathepsin B secretion to enhance axon outgrowth. J. Neurosci 38, 5399–5414. 10.1523/JNEUROSCI.3214-17.2018. 29760175 PMC5990985

[R87] Tran AP, Warren PM, and Silver J (2018b). The biology of regeneration failure and success after spinal cord injury. Physiol. Rev 98, 881–917. 10.1152/physrev.00017.2017. 29513146 PMC5966716

[R88] Tran AP, Warren PM, and Silver J (2022). New insights into glial scar formation after spinal cord injury. Cell Tissue Res. 387, 319–336. 10.1007/s00441-021-03477-w. 34076775 PMC8975767

[R89] Turcato F, Kim P, Barnett A, Jin Y, Scerba M, Casey A, Selman W, Greig NH, and Luo Y (2018). Sequential combined treatment of pifithrin-alpha and posiphen enhances neurogenesis and functional recovery after stroke. Cell Transplant. 27, 607–621. 10.1177/0963689718766328. 29871513 PMC6041885

[R90] Wiersma AM, Fouad K, and Winship IR (2017). Enhancing spinal plasticity amplifies the benefits of rehabilitative training and improves recovery from stroke. J. Neurosci 37, 10983–10997. 10.1523/JNEUROSCI.0770-17.2017. 29025926 PMC6596489

[R91] Yamada J, Nadanaka S, Kitagawa H, Takeuchi K, and Jinno S (2018). Increased synthesis of chondroitin sulfate proteoglycan promotes adult hippocampal neurogenesis in response to enriched environment. J. Neurosci 38, 8496–8513. 10.1523/JNEUROSCI.0632-18.2018. 30126967 PMC6596167

[R92] Yan YP, Sailor KA, Lang BT, Park SW, Vemuganti R, and Dempsey RJ (2007). Monocyte chemoattractant protein-1 plays a critical role in neuroblast migration after focal cerebral ischemia. J. Cereb. Blood Flow Metab 27, 1213–1224. 10.1038/sj.jcbfm.9600432. 17191078

[R93] Zhang R, Zhang L, Zhang Z, Wang Y, Lu M, Lapointe M, and Chopp M (2001). A nitric oxide donor induces neurogenesis and reduces functional deficits after stroke in rats. Ann. Neurol 50, 602–611. 11706966 10.1002/ana.1249

[R94] Zhang RL, Zhang Z, Zhang L, Wang Y, Zhang C, and Chopp M (2006). Delayed treatment with sildenafil enhances neurogenesis and improves functional recovery in aged rats after focal cerebral ischemia. J. Neurosci. Res 38, 1213–1219. 10.1002/jnr.20813 16511865

[R95] Zhang R, Zhang Z, and Chopp M (2016). Function of neural stem cells in ischemic brain repair processes. J. Cereb. Blood Flow Metab 36, 2034–2043. 10.1177/0271678X16674487. 27742890 PMC5363673

[R96] Zhang Y, Roos M, Himburg H, Termini CM, Quarmyne M, Li M, Zhao L, Kan J, Fang T, Yan X, (2019). PTPsigma inhibitors promote hematopoietic stem cell regeneration. Nat. Commun 10, 3667. 10.1038/s41467-019-11490-5. 31413255 PMC6694155

