# Supplementary material for: Inhibition of CSPG receptor PTPσ promotes migration of newly born neuroblasts, axonal sprouting, and recovery from stroke
Source: Cell Rep. Author manuscript; Available in PMC 2022 Nov 21. (PMC9677607; doi:10.1016/j.celrep.2022.111137)
Supplement: 1 [file NIHMS1829254-supplement-1.pdf]

Cell Reports, Volume *40*

Supplemental information

Inhibition of CSPG receptor PTPσ promotes migration of newly born neuroblasts, axonal sprouting, and recovery from stroke

Fucheng Luo, Jiapeng Wang, Zhen Zhang, Zhen You, Alicia Bedolla, FearGod Okwubido-Williams, L. Frank Huang, Jerry Silver, and Yu Luo

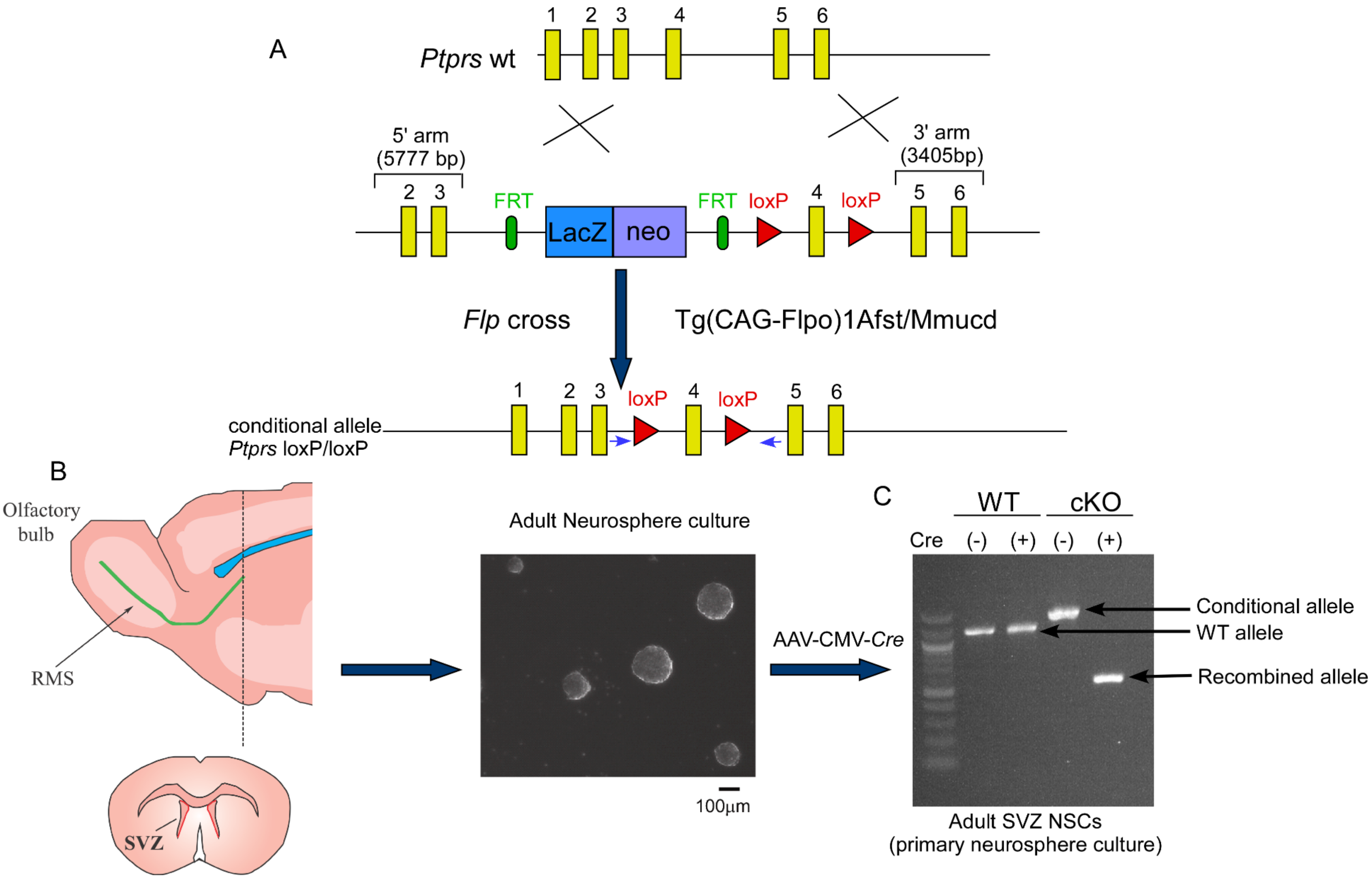

Fig S1. Generation of the *Ptprs* loxP/loxP mice (exon 4 is flanked by two loxP sites). Related to Fig 2. (A). Generation of adult NSC neurosphere cultures from the *Ptprs* loxP/loxP mice (B). Deletion of the *Ptprs* gene is achieved by infection of AAV-CMV-cre one passage before the specific assay. Confirmation of deletion of exon 4 by PCR primers flanking the two loxP sites (C).

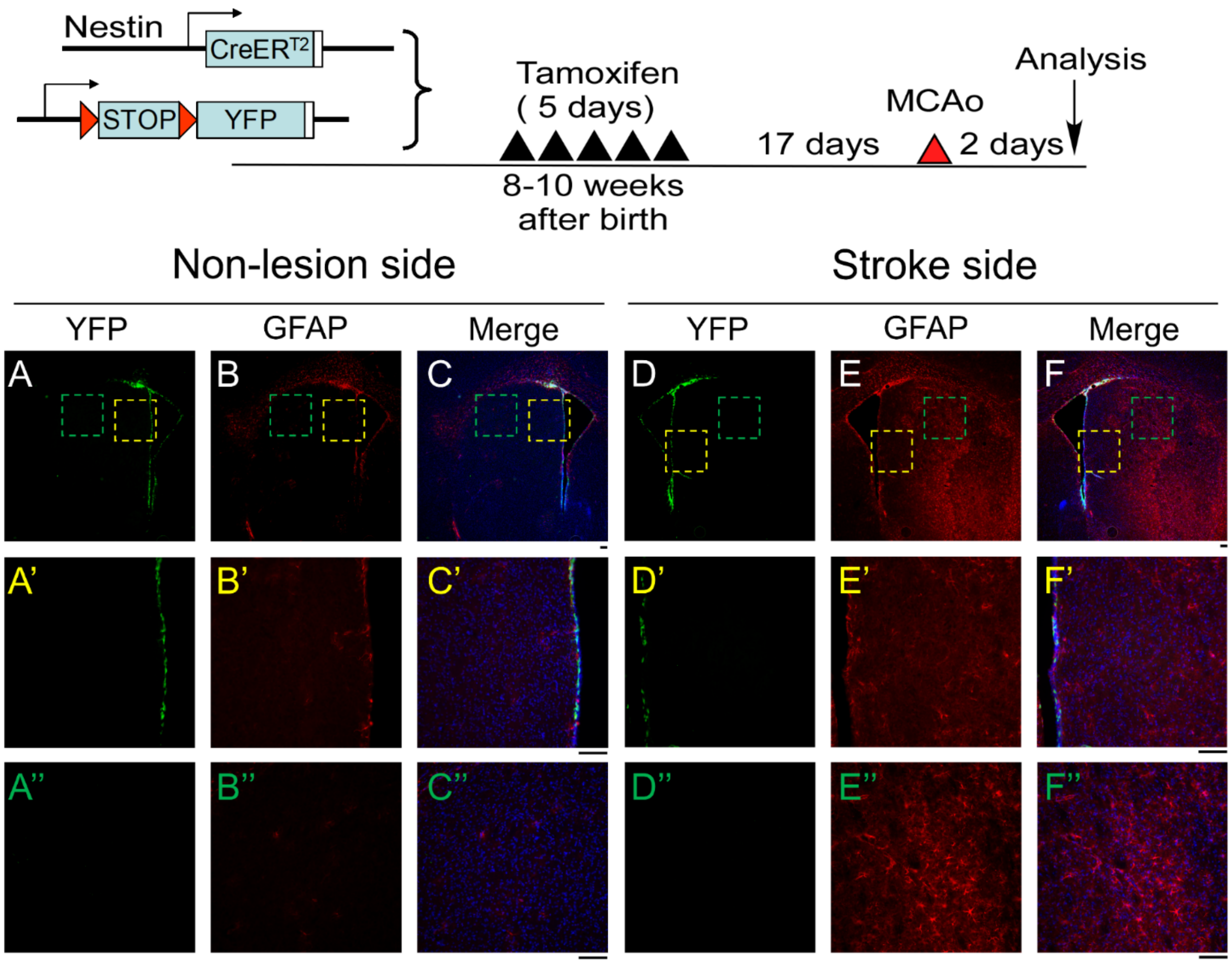

Fig S2 NestincreER-ROSA26YFP in mice does not label reactive astrocytes at 2 days post stroke when a regimen of Tamoxifen was given 17 days before stroke. Relate to Fig 3. YFP+ cells were detected in both the non-lesion side and stroke side SVZ (A' and D') at 2 days after stroke. However, although NESTIN/GFAP are upregulated in reactive astrocytes (F''), there is no YFP expression in GFAP+ reactive astrocytes in the stroke side striatum or cortex (D''), indicating successful clearing of Tamoxifen from the brain and specific labeling of SVZ NSCs and their progeny in this treatment paradigm. Scale bar =100um. Similar results are observed in n=3 mice.

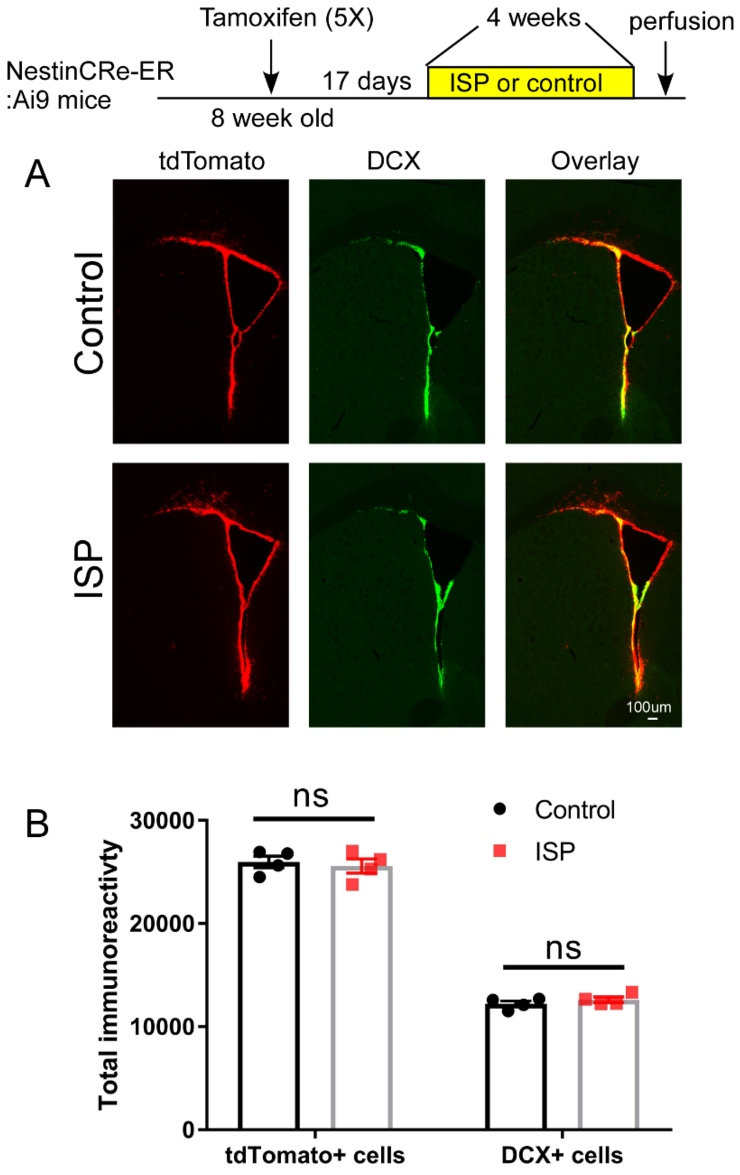

Fig S3. 30 day ISP treatment in non-stroke mice show similar tdTomato+ and DCX+ cells at the SVZ. Related to Fig 3. Quantification shown in (B). Mean $\pm$ SEM. Multiple sections at similar locations were analyzed for each animal and average immunoreactive intensity for each mouse was presented as an individual dot on the plot and analyzed as a single data point for statistical analysis.

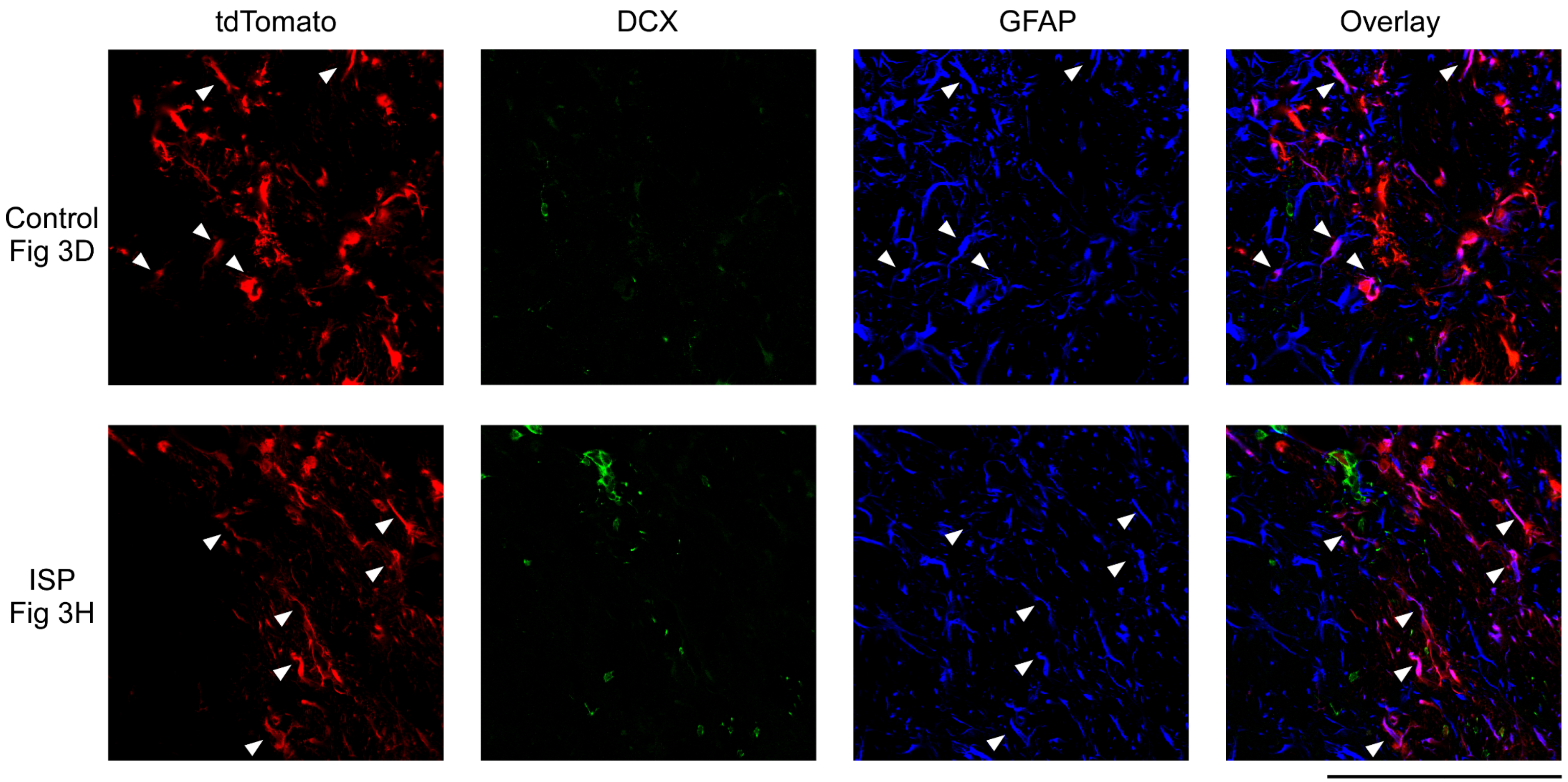

Fig S4. Single Channel images for Fig 3 panel D and H showing differentiation of tdTomato+ NSCs into GFAP+ astrocytes. Related to Fig 3. Scale bar=100um.

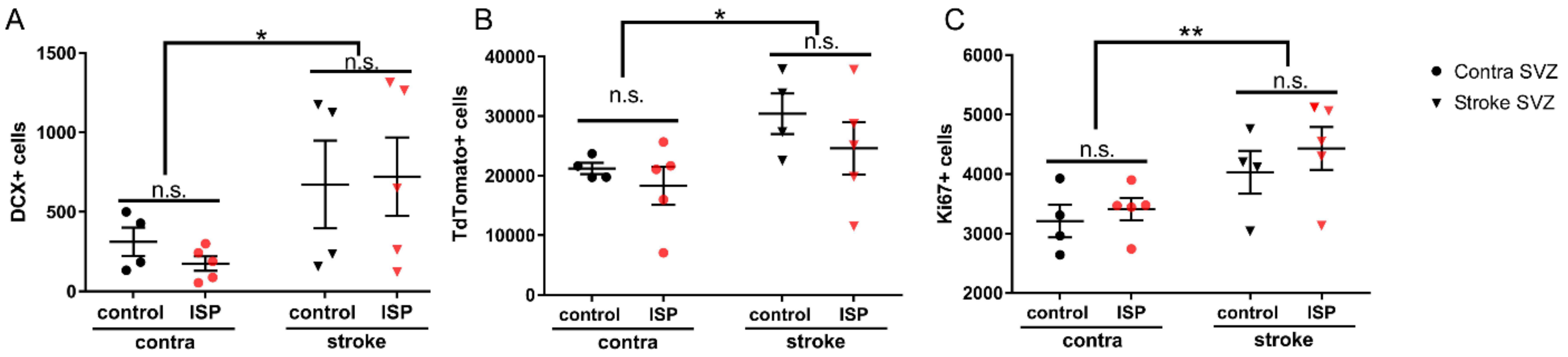

Fig S5. ISP treatment starting at psd1 does not change the number of proliferating cells (Ki67+), immature neuroblasts (DCX+) or total newly born cells at 14 days post-stroke within the SVZ (A-C). Related to Fig 3. Mean + SEM. Multiple sections at similar locations were analyzed for each animal and average immunoreactive intensity for each mouse was presented as an individual dot on the plot and analyzed as a single data point for statistical analysis. *$p<0.05$, ** $p<0.01$, ns, $p > 0.05$.

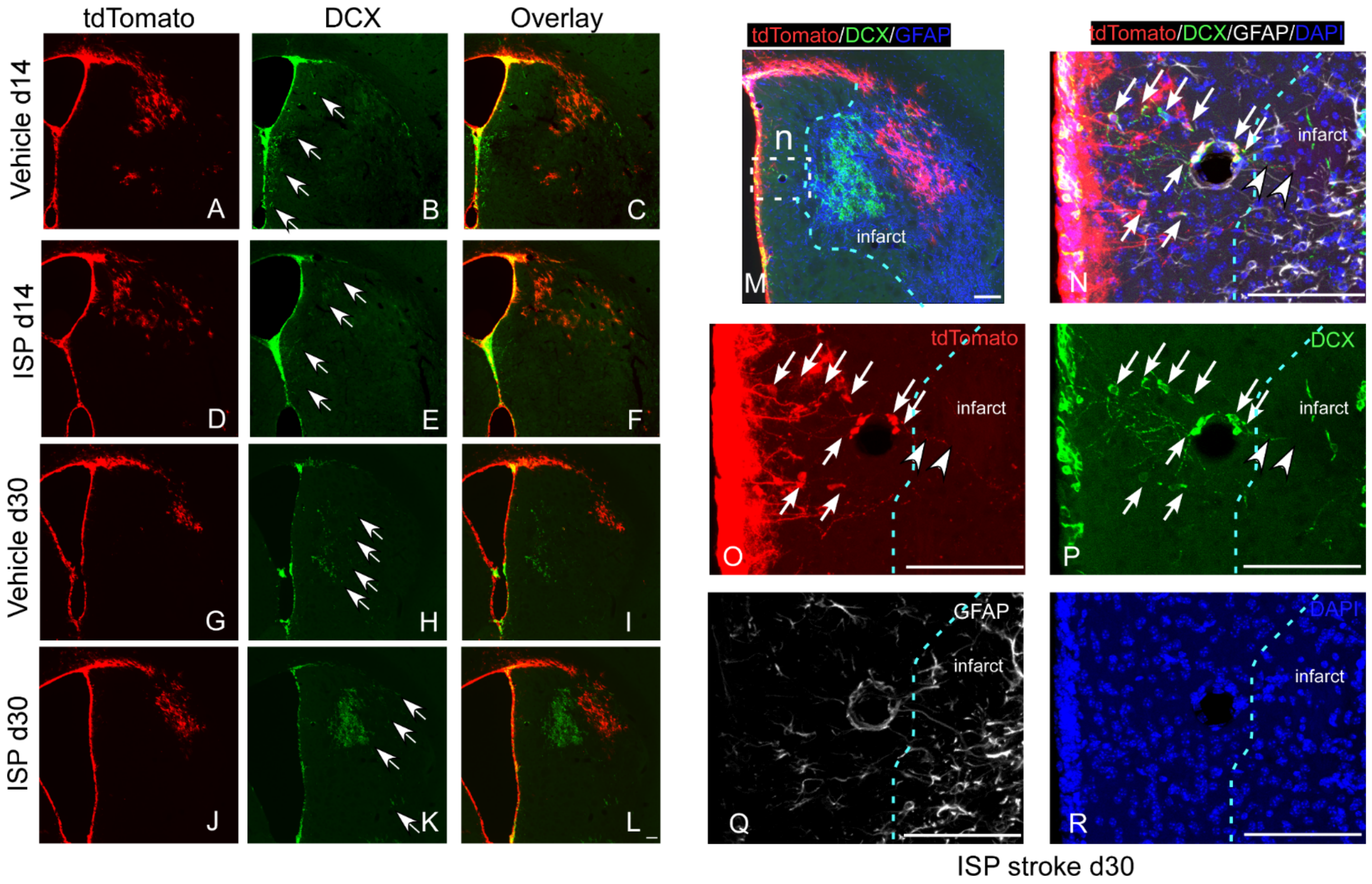

Fig S6. Migration of neuroblasts at high magnification. Related to Fig 3. **(A-L)** Migration of SVZ tdTomato positive or DCX+ neuroblasts into the infarct region from d14 to d30 after stroke. White arrows in B, E, H, K indicate the front of DCX+ cells migrating into the infarct zone. **(M-R).** Representative migratory chains of tdTomato+/DCX+ cells from the SVZ towards the infarct zone. Note that tdTomato+/DCX+ are associated with GFAP+ astrocytic endfeet covering blood vessels, a migratory pattern of neuroblasts that has been previously described in stroke brain (Jin et al., 2003; Ohab et al., 2006). Scale bar =100um.

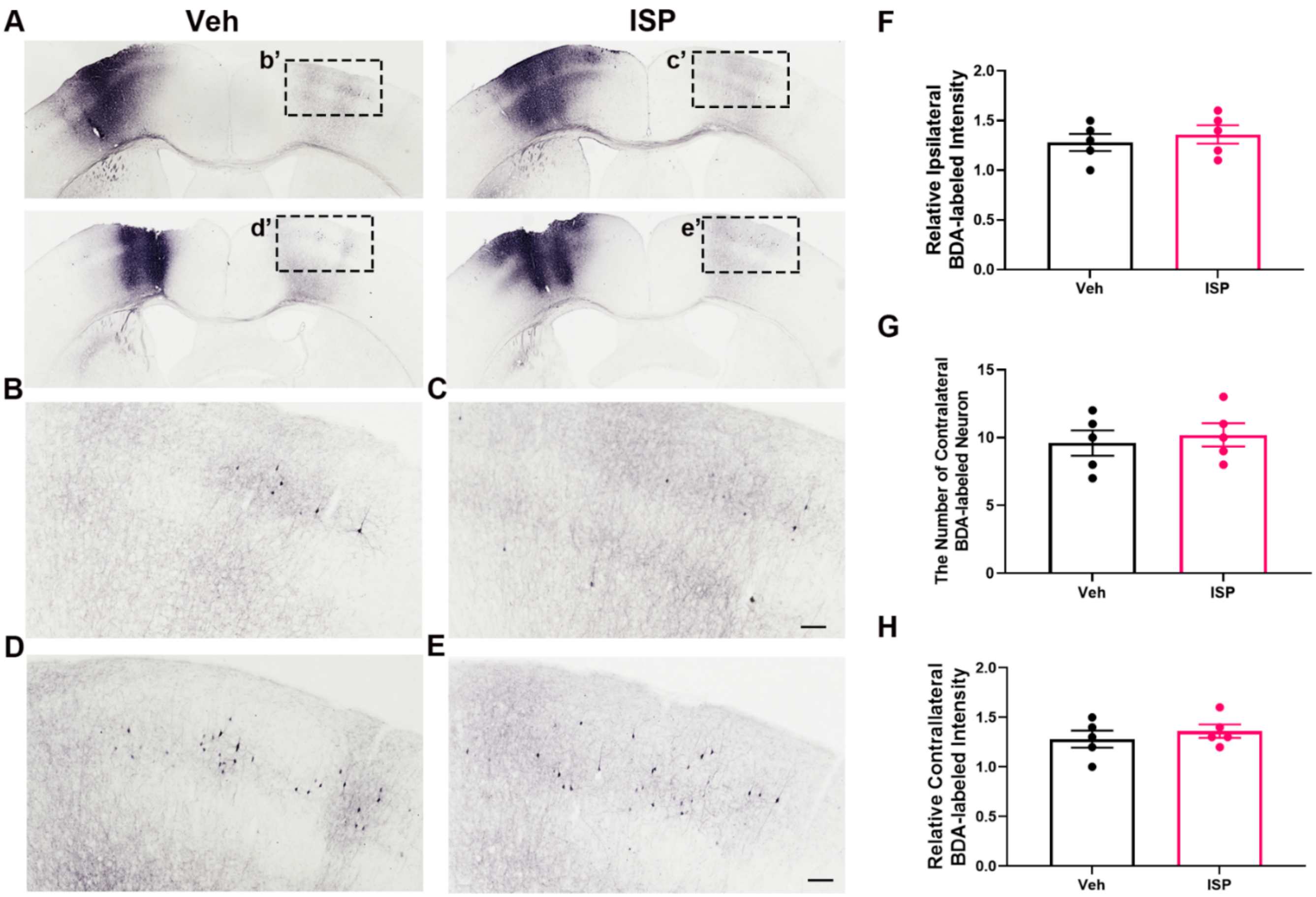

Fig S7. ISP treatment in non-stroke mice does not affect BDA uptake and retrograde or anterograde labeling. Related to Fig 4. Injection of BDA in one side of cortex in non-stroke mice labels axons in the contralateral cortex as well as retrogradely labels some neuron cell bodies in the contralateral cortex. Daily ISP treatment for 4 weeks in 3 month old mice (BDA injected at 2weeks after initiation of ISP treatment) does not affect the number of retrogradely labeled neuronal cell bodies or fiber density in the contralateral side (2 representative coronal positions shown for Veh or ISP treated mice. $p>0.05$ for all measurements, Student's t-test). Scale bar=100µm.

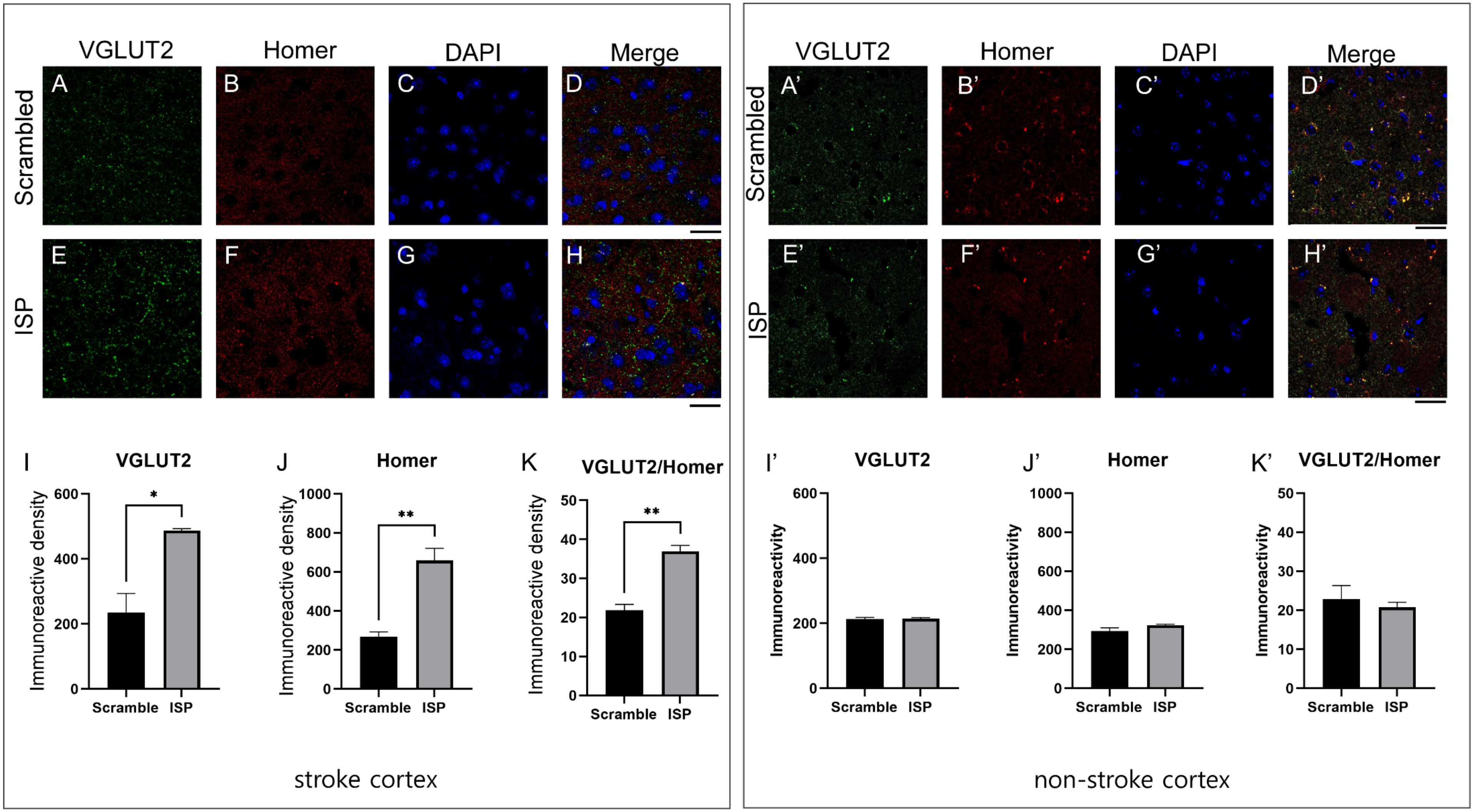

Fig S8. Related to Figs 4 and 5. Post-stroke ISP treatment (psd1-psd 30) increases synaptic density at the peri-infarct zone measured by the pre-synaptic marker VGlut2 and post-synaptic marker Homer (A-K). ISP treatment (for 30 days) in non-stroke mice does not affect synaptic density in cortex measured by the pre-synaptic marker VGlut2 and post-synaptic marker Homer (A'-K'). Three images from three sections encompassing the infarct core were quantified from each animal and the average from each mouse was used as a single data point for statistical analysis. Mean $\pm$ SEM. n= 3 mice for each group. *$p<0.05$, ** $p<0.01$, Student's t-test.

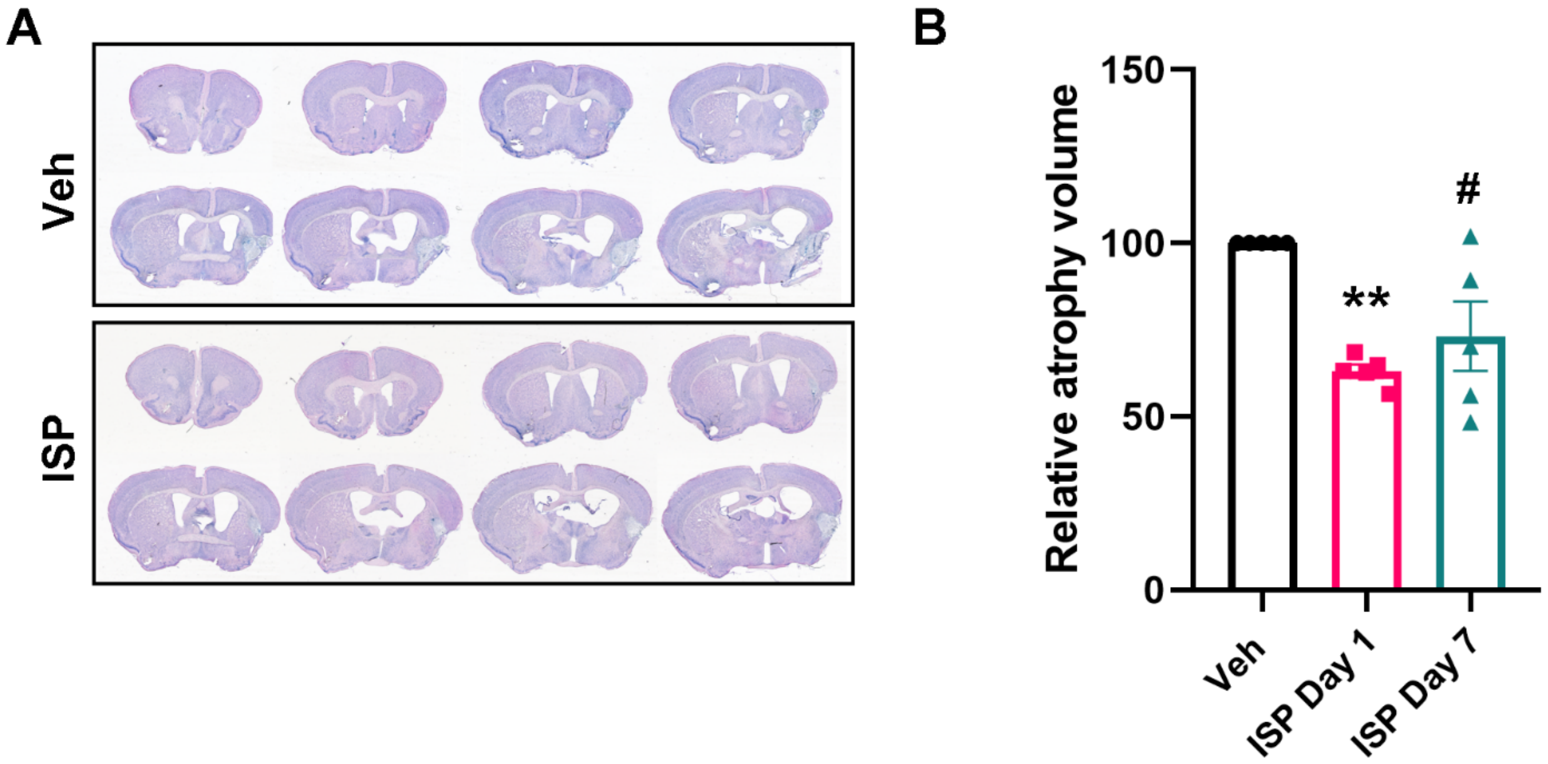

Fig S9. ISP treatment decreased brain atrophy after stroke. Related to Figs 4 and 5. ISP treatment started at psd1 resulted in the least atrophy compared to vehicle (Veh) mice. ISP treatment started at psd7 also show reduced atrophy but to a less extent compared to psd1 treatment and shows bigger variations among animals compared to Veh (A,B). **, $p<0.01$ and #, $p<0.05$, ANOVA.

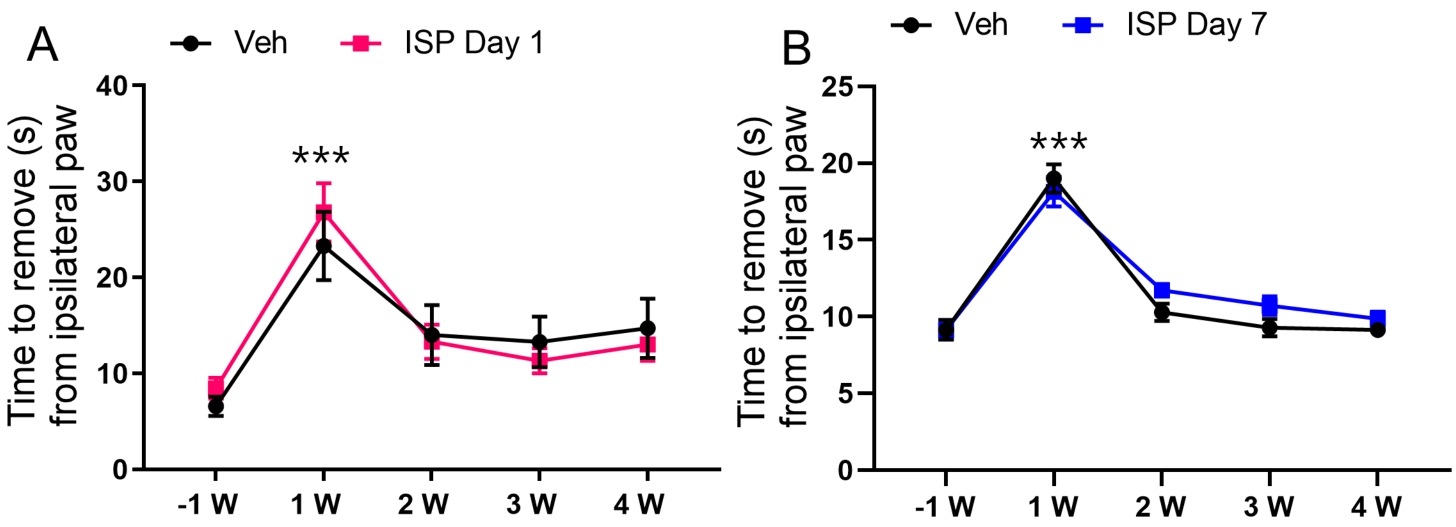

Fig S10. For the Adhesive removal test, post-stroke ISP treatment starting at day 1 or day 7 does not affect the time to remove the tape from the unaffected ipsilateral side (left paw in our model). Related to Figs 6 and 7. Note that the time to remove the tape transiently increases for the 1-week post-stroke time point (*** compared to pre-stroke baseline ANOVA), but quickly returns to pre-stroke levels at later time points with no differences between Veh and ISP groups. (n=7 for Veh and n=12 for ISP for ISP Day 1 treatment paradigm and n=7 for Veh and n=7 for ISP for ISP Day 7 treatment paradigm).

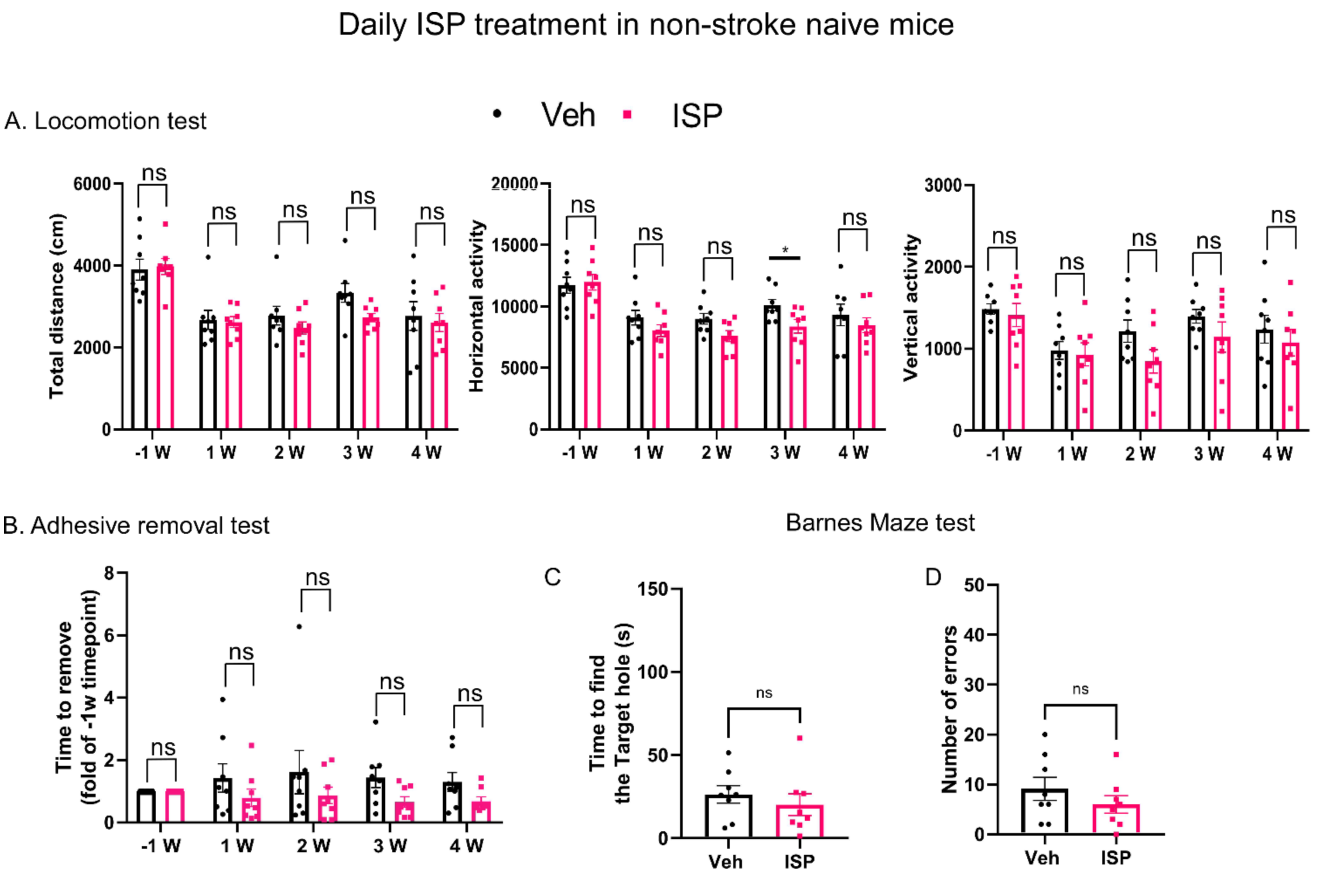

Fig S11. The effects of daily ISP treatment on the non-stroke naïve mice at different time points after ISP treatment. Related to Figs 6 and 7. -1w (week) data were baseline data measured before the treatment of Veh or ISP (no baseline data was obtained for the Barnes Maze test to avoid memory from the baseline test). General locomotor performance was measured by automated open field chambers for 1 h (A). Fine motor function was measured by the adhesive tape removal test (B) Cognitive function was measured by Barnes Maze at 4 w after stroke (C-D). * $p<0.05$, Two-way RM ANOVA for (A-B) and Student's t-test for (C-D). Each data point represents an individual mouse.

Table S3. Effect Size and p value from RM Two Way ANOVA for behavioral analysis. Related to Figs 6 and 7.

ISP day 1

Total distance	Veh Mean	ISP Mean	Veh SD	ISP SD	Cohen's d	Coefficient, r	P value ISP vs Veh
-1 W	2568.57143	2656	366.8973	450.341688	0.212854169	0.102138847	0.657
3 d	1196.42857	1154.083	380.1477	488.320397	-0.096769325	-0.046628465	0.829
1 W	1439.28571	1666.417	383.2887	486.072471	0.518909691	0.242818449	0.25
2 W	1793.42857	2317.083	340.6713	448.911706	1.314118081	0.535393349	0.009
3 W	1916.28571	2553.167	266.8418	366.076205	1.988232959	0.692184948	0.002
4 W	2136.14286	2677.417	229.2949	443.651677	1.532784907	0.594520436	0.007
Horizontal activity							
-1 W	9208.85714	10238	938.6168	1616.01119	0.778794452	0.351674943	0.119
3 d	5141.71429	5181.417	1607.212	1100.29545	0.028826733	0.013903991	0.952
1 W	7081.28571	7030.583	1870.861	1365.6217	-0.030956849	-0.014931189	0.938
2 W	7771.71429	8878	961.2668	1520.2019	0.86984517	0.386913257	0.094
3 W	8212.71429	10024.75	977.5199	1339.79463	1.545142131	0.597606104	0.007
4 W	9091.71429	10957.08	932.3379	1545.03165	1.461882421	0.576298702	0.006
Vertical activity							
-1 W	1082.71429	1244.333	246.0696	229.753359	0.678925285	0.311232021	0.133
3 d	296.285714	278.0833	261.8834	243.544082	-0.071980301	-0.034700686	0.865
1 W	525.142857	753.3333	183.4189	206.257268	1.169173859	0.491241431	0.035
2 W	772.571429	988.9167	190.2734	225.805813	1.036152017	0.447081398	0.046
3 W	789.857143	1054.583	124.6026	208.274058	1.542552341	0.596961605	0.015
4 W	904.285714	1211.75	146.5853	297.312852	1.311734861	0.534700168	0.005
Adhesive removal							
-1 W	12.2857143	9.166667	5.82278	6.19139187	-0.51898387	-0.242851113	0.682
1 W	113.428571	99.33333	12.13613	18.5978168	-0.897617911	-0.397341921	0.067
2 W	94.5714286	72.5	11.74531	25.6957726	-1.104797669	-0.470309933	0.005
3 W	84.8571429	52.25	9.370674	23.2540319	-1.839308333	-0.663674096	<0.001
4 W	63.1428571	36.33333	7.515064	11.0068849	-2.844780385	-0.808176426	<0.001
Barnes maze							
latency	92.7142857	62.16667	14.69086	19.6090579	-1.763173137	-0.647875297	0.002393638
error trials	10.7857143	4.291667	2.233404	2.61515108	-2.670491141	-0.789922215	3.983E-05

**For Cohen's d**		**For Coefficient, r**	
Relative size	Effect size	Strength of Association	Effect size
small	0.2-0.5	small	0.-0.3
medium	0.5-0.8	medium	0.3-0.5
large	>0.8	large	>0.5-1

ISP day 7

	Veh Mean	ISP Mean	Veh SD	ISP SD	Cohen's d	Coefficient, r	P value ISP vs Veh
Total distance							
-1 W	3545.14286	3405.143	528.5604	701.065719	-0.225503176	-0.112041649	0.626
1 W	1600.71429	1864	750.5435	653.337075	0.374186856	0.183902456	0.36
2 W	1855.42857	2551.714	531.426	585.112443	1.245782806	0.528711296	0.018
3 W	1903.14286	2593.286	422.0527	373.040978	1.732715016	0.654797091	0.019
4 W	2280.28571	3057	144.606	349.147533	2.906628473	0.823817858	0.009
Horizontal activity							
-1 W	11227.1429	11671.29	2325.659	2024.42739	0.203711749	0.101331592	0.696
1 W	6708.71429	7269.143	1831.069	3041.24357	0.223262596	0.110942183	0.622
2 W	8343.14286	10025.57	2385.363	1824.65658	0.792254182	0.368284554	0.144
3 W	8268.42857	10452.86	2280.207	1889.35725	1.043223702	0.462477301	0.06
4 W	7641	11435.29	1612.843	1408.82726	2.505678647	0.781559554	0.002
Vertical activity							
-1 W	1319.42857	1548.429	325.3587	372.445458	0.654855413	0.311172093	0.205
1 W	401.857143	588.5714	304.0041	343.89381	0.575280189	0.276431779	0.298
2 W	598.714286	853.1429	394.8256	256.851727	0.763908472	0.35681249	0.16
3 W	624.571429	918.4286	368.0688	314.748501	0.858107391	0.39429369	0.108
4 W	593.857143	1091	305.3503	263.823173	1.74226289	0.656850932	0.01
Adhesive removal							
-1 W	8	9.142857	2.081666	4.29839394	0.338414401	0.166835712	0.914
1 W	108.142857	112.5714	25.37997	19.6541526	0.195105587	0.097091898	0.674
2 W	98.1428571	78.71429	23.56147	26.2406446	-0.779104217	-0.362983003	0.072
3 W	79.5714286	55	26.77597	21.023796	-1.020734065	-0.454585501	0.025
4 W	49.8571429	21.85714	18.24307	5.89995964	-2.065256636	-0.71836491	0.011
Barnes maze							
latency	138.571429	54.92857	70.30969	29.8725467	-1.548434575	-0.612184929	0.013404364
error trials	24	7.857143	13.87143	4.32737686	-1.571112212	-0.61774464	0.012388494

Note: Red letters indicate that the effect size that also have statistical significance ($p<0.05$ by post-hoc analysis between veh and ISP in each time point).

Table S4. Time point comparison to baseline level within Veh or ISP group. Related to Figs 6 and 7.

	**Veh group** each time point vs baseline *	**ISP group** each time point vs baseline *
Total distance		
-1 W	N/A	N/A
3 d	<0.001	<0.001
1 W	<0.001	<0.001
2 W	0.001	>0.05
3 W	0.013	>0.05
4 W	>0.05	>0.05
Horizontal activity		
-1 W	N/A	N/A
3 d	<0.001	<0.001
1 W	0.015	<0.001
2 W	>0.05	>0.05
3 W	>0.05	>0.05
4 W	>0.05	>0.05
Vertical activity		
-1 W	N/A	N/A
3 d	<0.001	<0.001
1 W	<0.001	<0.001
2 W	0.064	0.032
3 W	>0.05	>0.05
4 W	>0.05	>0.05
**Adhesive removal**		
-1 W	N/A	N/A
1 W	<0.001	<0.001
2 W	<0.001	<0.001
3 W	<0.001	<0.001
4 W	<0.001	<0.001

* host-hoc P value for RM ANOVA
